# Benchmarking cancer outcomes in Europe: a scoping review of methodologies and case-mix adjustments

**DOI:** 10.1016/j.esmorw.2025.100176

**Published:** 2025-09-03

**Authors:** J. Thurell, L. Doppelbauer, E.M. Verheul, I. Petrov, M.M. Karsten, L.B. Koppert, J. Bergh, I. Fredriksson, P. Lindgren, N. Kiani, E. Hedayati

**Affiliations:** 1 Department of Oncology-Pathology, Karolinska Institutet, Solna, Sweden; 2 Breast Cancer Center, Department of Breast, Endocrine Tumours and Sarcoma, Karolinska Comprehensive Cancer Center, Karolinska University Hospital, Stockholm, Sweden; 3 Department of Gynecology with Breast Center, Charité – Universitätsmedizin Berlin, Campus Virchow-Klinikum, Berlin, Germany; 4 Department of Public Health, Centre for Medical Decision Making, Erasmus University Medical Centre, Rotterdam, The Netherlands; 5 Department of Microbiology, Tumor- and Cell Biology, Karolinska Institutet, Solna, Sweden; 6 Cancer Centrum Karolinska, Stockholm, Sweden; 7 Division of Medical Diagnostics, Radiology, Karolinska University Hospital C1:46, Stockholm, Sweden; 8 Department of Surgery, Erasmus MC Cancer Institute, Rotterdam, The Netherlands; 9 Department of Molecular Medicine and Surgery, Karolinska Institutet, Karolinska University Hospital, (L1:00), Stockholm, Sweden; 10 Department of Learning, Informatics, Management and Ethics, Karolinska Institutet, Stockholm, Sweden; 11 Algorithmic Dynamics Lab, Center for Molecular Medicine, Karolinska Instiutet, Solna, Sweden

**Keywords:** health care, quality indicators, benchmarking, risk adjustment, neoplasms, systematic review, Europe

## Abstract

**Background:**

Benchmarking hospital outcomes is crucial for identifying inequities and improving cancer care. Meaningful comparisons require selecting relevant outcomes and adjusting for case-mix factors such as age, comorbidity, and stage. Without case-mix adjustment, hospitals may be unfairly assessed based on patient mix rather than care quality. No prior review has examined benchmarking practices in European cancer care. This scoping review addresses: (i) Which health outcomes are frequently benchmarked? (ii) What case-mix factors are commonly used for adjustment? (iii) Which statistical approaches are utilized? (iv) How are case-mix models developed and evaluated?

**Materials and methods:**

We conducted a systematic scoping review searching OVID MEDLINE, Web of Science, and EMBASE. Eligible studies focused on benchmarking populations with a cancer diagnosis, involved European hospitals, and evaluated health outcomes like survival. Abstract screening and full-text appraisal were done independently by two authors. Data were extracted into a pre-specified matrix, and results synthesized by research question.

**Results:**

After screening 4953 abstracts, 65 studies were included. Key gaps include a lack of validated case-mix models, under-representation of long-term outcomes, and a tendency to ‘over-adjust’ by including treatment factors in case-mix models, potentially obscuring true differences in performance. Regression modeling remains the gold standard for adjustment. A consensus is needed on reporting and evaluating case-mix models, akin to TRIPOD guidelines.

**Conclusions:**

A shift toward standardized, validated benchmarking practices is essential to drive health care improvements. Only through rigorous methodologies, standardized reporting, and international collaboration can hospital benchmarking become a transformative tool for improving cancer care quality and patient outcomes.

## Highlights

• Cancer benchmarking lacks clear standards on methods for case-mix adjustment.
• Benchmarking is concentrated in two countries, limiting learning across Europe.
• Lack of long-term survival and patient-reported outcomes in benchmarking studies.
• Case-mix models lack validation and clear reporting, undermining fair comparisons.
• We propose practical guidance on case-mix modeling to support fair benchmarking.

## Introduction

In many countries there is a growing interest in assessing health care quality and equity. Over the past few decades, benchmarking has emerged as a vital tool to address these demands. By systematically comparing performance among health care institutions, benchmarking identifies best practices and drives improvements in underperforming hospitals.^1^ In the rapidly evolving field of cancer care, continuously monitoring real-world outcomes is essential to ensure all patients receive optimal treatment, regardless of the hospital they attend. However, benchmarking must be done carefully. Inappropriate practices, especially when results are publicly reported, risk drawing misleading conclusions that can unfairly harm hospitals and erode public trust in health care systems.

A key challenge in benchmarking is determining which quality metrics to measure. Although process indicators assess specific care steps and guideline adherence, outcomes provide a more comprehensive, patient-centered view of health care performance by capturing the effectiveness of the entire care process.^2^ However, benchmarking outcomes require careful consideration of confounding factors. Hospitals serve diverse patient populations with varying characteristics (i.e. case-mix) related to age, disease severity, and socioeconomic factors influencing outcomes. Without robust statistical adjustments for these factors—over which hospitals have no control—benchmarking may yield unfair comparisons that distort the accurate picture of performance.^3^

Despite the growing use of benchmarking, no review has systematically examined its application in European cancer care. This scoping review aims to describe how benchmarking of cancer outcomes has been applied in European countries, specifically addressing:

• Which health outcomes are most frequently benchmarked?
• What case-mix factors are commonly used for adjustment?
• Which statistical approaches are utilized for case-mix adjustment?
• How are case-mix models developed, and quality assessed?

Effective benchmarking is essential for ensuring high-quality and equitable cancer care. If poorly implemented, it risks compromising the clinical pathways and care processes it is designed to enhance. This review aims to provide a foundation for more accurate and transparent benchmarking practices that can transition successfully to real-world applications.

## Methods

### Search strategy and selection criteria

This scoping review was conducted in accordance with the PRISMA guidelines.^4^ Studies were eligible if they focused on benchmarking populations with a cancer diagnosis, involved European hospitals or regions, and evaluated health outcomes (e.g. survival, patient-reported outcomes, or post-operative complications). This review defined Europe as the 27 member states of the European Union (EU), along with Norway, the UK and Switzerland, given their comparable health care systems, and applying case-mix adjustments (e.g. regression modeling and advanced machine learning). Studies comparing variations between hospitals or cancer networks/regions within countries were included. To ensure the inclusion of rigorous research, we included peer-reviewed studies only, published in English, Swedish, Dutch, or German before 11 November 2024. Studies were ineligible if their models aimed solely at individual prediction as this review focused on case-mix adjusted models designed to compare the quality of care across health care providers rather than to predict individual patient outcomes. Studies focusing exclusively on process indicators or conducted outside Europe were excluded, to limit the scope of the review. Cross-sectional studies were also excluded as we wanted to track outcomes longitudinally. Finally, we excluded studies without case-mix adjustment, as assessing case-mix adjustment methods is a key aim of this review. We searched OVID MEDLINE, Web of Science, and EMBASE using structured search terms related to ‘cancer,’ ‘case-mix adjustment,’ and ‘benchmarking.’ The full search strings are presented in Supplementary Appendix 1, available at https://doi.org/10.1016/j.esmorw.2025.100176.

### Data analysis

Two reviewers independently screened titles and abstracts using Rayyan.ai, resolving conflicts through discussion or consultation with a third reviewer. Full-text screening followed the same procedure. Studies excluded after full-text screening are described in Supplementary Appendix 2, available at https://doi.org/10.1016/j.esmorw.2025.100176. For quality appraisal, we applied the Newcastle-Ottawa Scale^5^ for cohort studies and incorporated elements from the Joanna Briggs Institute (JBI) scale^6^ to evaluate the robustness of statistical methods. No studies were excluded based on the quality appraisal but instead contributed to synthesizing the results. The full quality appraisal is described in Supplementary Appendix 3, available at https://doi.org/10.1016/j.esmorw.2025.100176. Data were extracted using a predefined matrix based on the JBI framework for scoping reviews,^7^ focusing on population, intervention, and context (Supplementary Appendix 4, available at https://doi.org/10.1016/j.esmorw.2025.100176). Results were synthesized according to predefined research questions.

### Role of funding source

The funder of the study had no role in study design, data collection, data analysis, data interpretation, or writing of the report.

## Results

**Figure 1:**
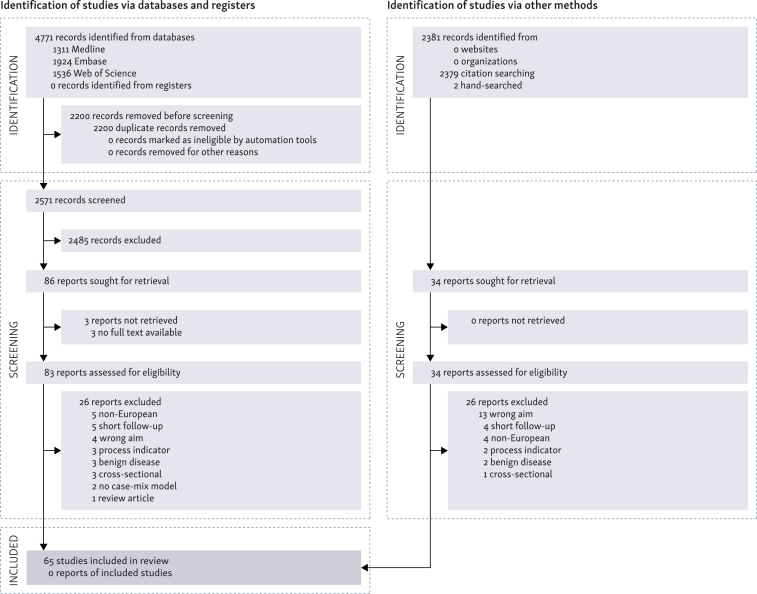
Study selection.

We screened 2571 articles based on title and abstract. Eighty-three articles were retrieved in full text, and 57 met the eligibility criteria. We also screened all included articles’ reference lists and hand searched for additional articles. This resulted in 8 further added articles, summing to a total of 65 articles^8^, ^9^, ^10^, ^11^, ^12^, ^13^, ^14^, ^15^, ^16^, ^17^, ^18^, ^19^, ^20^, ^21^, ^22^, ^23^, ^24^, ^25^, ^26^, ^27^, ^28^, ^29^, ^30^, ^31^, ^32^, ^33^, ^34^, ^35^, ^36^, ^37^, ^38^, ^39^, ^40^, ^41^, ^42^, ^43^, ^44^, ^45^, ^46^, ^47^, ^48^, ^49^, ^50^, ^51^, ^52^, ^53^, ^54^, ^55^, ^56^, ^57^, ^58^, ^59^, ^60^, ^61^, ^62^, ^63^, ^64^, ^65^, ^66^, ^67^, ^68^, ^69^, ^70^, ^71^, ^72^ included in the review (Figure 1), and a total of 1 304 433 cases from eight European countries (Supplementary Appendix 4, available at https://doi.org/10.1016/j.esmorw.2025.100176). Fifty-two studies (80%) were conducted in either the Netherlands or the UK, whereas the remaining 13 studies (20%) represented other European countries. Colorectal cancer was the most commonly benchmarked cancer type, accounting for 31% of included studies, followed by head and neck cancers and lung cancer (Table 1).

**Table 1:** Summary of the included studies

Category	Subcategory	Studies, *n* (%)
Countries		
	The Netherlands	29 (44.5)
	United Kingdom	23 (35)
	France	3 (5)
	Germany	3 (5)
	Norway	2 (3)
	Sweden	2 (3)
	Denmark	1 (1.5)
	Austria	1 (1.5)
	European collaborations	1 (1.5)
Cancer types		
	Colorectal	20 (31)
	Head and neck	9 (14)
	Lung	9 (14)
	Gastroesophageal	6 (9)
	Breast	6 (9)
	Liver metastasis	5 (8)
	Gynecological	5 (8)
	Skin	1 (1.5)
	Brain	1 (1.5)
	Leukemia	1 (1.5)
	Prostate	1 (1.5)
	Several cancer types	1 (1.5)
Case-mix factors		
	Basic demographics	65 (100)
	Tumor factors	54 (83)
	Patient factors	51 (78)
	Socioeconomic factors	14 (22)
	Treatment factors	39 (60)

**Figure 2:**
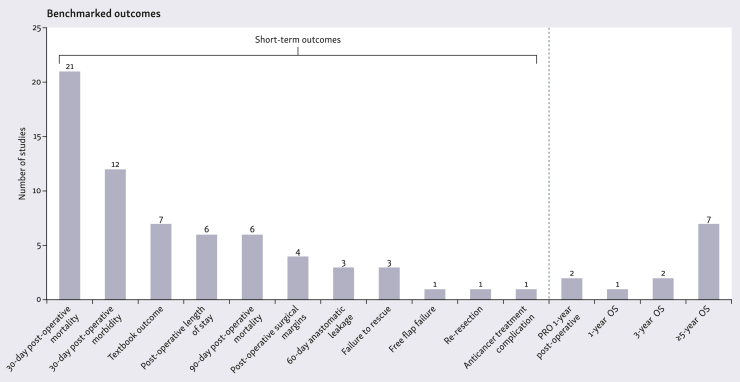
Outcomes that were benchmarked in the included studies^a^. ^a^Some studies benchmarked more than one outcome.

Seventy-seven outcomes were benchmarked across the 65 included studies. Fifty-three studies (82%) benchmarked short-term outcomes occurring within 90 days of surgery and/or oncological treatment. The most commonly benchmarked short-term outcomes were 30-day post-operative mortality and 30-day post-operative morbidity. Seven studies benchmarked so-called ‘textbook outcomes’ (TOs). TO is a composite measure of process indicators and outcomes specific to each cancer type that reflect high-quality care. It is an all or none score for each patient. These typically include no death or severe complications within 30 days post-surgery, alongside other criteria such as no readmission and sufficient surgical margins. Failure to rescue (FTR) was used in three studies. FTR assesses mortality rates among patients who develop severe complications after major surgery (Figure 2).

Long-term outcomes (here defined as follow-up of at least 1 year) were benchmarked in 12 studies (18%) (Table 1). Of these, 10 studies benchmarked survival outcomes, whereas the remaining two studies benchmarked patient-reported outcomes (PROs) (Figure 2).

All studies accounted for either age or sex in their adjustments. Most studies also adjusted for tumor characteristics (e.g. stage) and patient characteristics (e.g. comorbidity burden, Eastern Cooperative Oncology Group performance status). Fourteen studies (22%) incorporated socioeconomic factors, primarily using country-specific social status indices based on postal codes.^10^^,^^15^^,^^27^^,^^29^^,^^30^^,^^32^^,^^36^^,^^39^^,^^41^^,^^42^^,^^46^, ^47^, ^48^^,^^70^ All but two of these studies were from the UK. Sixty percent of studies (*n* = 39) adjusted for treatment factors within hospital control.^8^^,^^10^, ^11^, ^12^, ^13^^,^^16^, ^17^, ^18^, ^19^, ^20^, ^21^, ^22^, ^23^^,^^25^^,^^26^^,^^31^^,^^33^, ^34^, ^35^, ^36^, ^37^, ^38^^,^^47^, ^48^, ^49^, ^50^^,^^54^, ^55^, ^56^, ^57^, ^58^, ^59^, ^60^^,^^62^, ^63^, ^64^^,^^66^^,^^67^^,^^72^ The most common treatment factors adjusted for were surgical complexity, surgical approach, and preoperative systemic treatment and/or radiotherapy (Table 1).

Of the 65 included studies, 18 (28%) failed to describe the content of their case-mix models. Furthermore, 61 studies (94%) developed new case-mix models, whereas the remaining 4 focused on externally validating previously established models. None of the four external validation studies^18^^,^^21^^,^^58^^,^^60^ succeeded in their validation as either discrimination or calibration differed significantly between the development and validation cohorts (Supplementary Appendix 4, available at https://doi.org/10.1016/j.esmorw.2025.100176).

**Figure 3:**
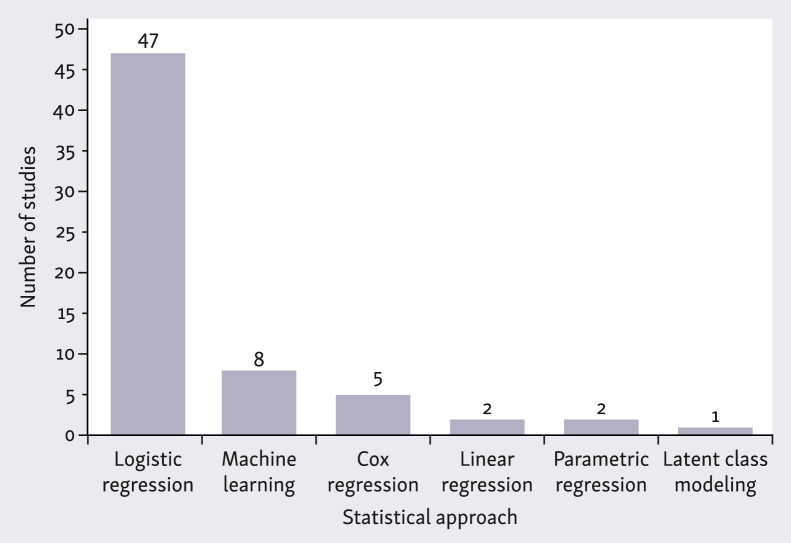
Distribution of the main statistical approach applied for case-mix adjustment in the included studies.

Classical regression models were the predominant approach for case-mix adjustment, serving as the primary method in 56 studies (86%). In studies applying regression models for short-term binary outcomes (up to 90 days), logistic regression was uniformly applied. In contrast, long-term survival analyses (spanning 1-10 years) exhibited greater methodological heterogeneity. Although Cox proportional hazards modeling was the most common approach—employed in five studies—other methods were also used, including parametric regression (*n* = 2),^32^^,^^48^ logistic regression for discrete survival benchmarks (*n* = 2),^36^^,^^40^ and multilevel latent class modeling (LCM) (*n* = 1).^30^ LCM, which groups patients into unobserved subgroups based on data patterns, yielded hospital rankings comparable with those obtained from traditional multilevel regression, but provided no in-depth comparative analysis between the two methods. Furthermore, linear regression was applied in two studies to benchmark PROs^39^^,^^47^ (Figure 3).

Machine learning (ML) methods, including techniques beyond traditional regression such as decision trees, random forests, and neural networks were applied in eight studies (13%), seven of them by the same research group (Tighe et al.). One of these compared logistic regression with three ML methods for adjusting post-operative complication rates after head and neck cancer surgery^54^ and found that a Naïve Bayes model (a probabilistic classifier) slightly outperformed traditional logistic regression in terms of discrimination [area under the curve (AUC) 0.72 versus 0.68]. The only study outside this research group benchmarked 30-day post-operative mortality rates in colorectal cancer, comparing logistic regression and three advanced ML techniques. Elastic net regression—a method that improves predictions by combining two regularization techniques (Least Absolute Shrinkage and Selection Operator, and ridge regression)—outperformed logistic regression by a small margin (AUC 0.82 versus 0.81) (Supplementary Appendix 4, available at https://doi.org/10.1016/j.esmorw.2025.100176).

Two primary strategies were used to assess the performance of case-mix models. The first involved visualizing the comparison between crude and adjusted outcomes. The second focused on statistical performance metrics, primarily discrimination (i.e. the model’s ability to distinguish between patients who experienced the outcome and those who did not) and calibration (i.e. the alignment between predicted and observed outcomes across different risk groups). Of the 65 studies, 18 neither visualized crude to adjusted outcomes, nor provided any statistical performance metrics. Fourteen studies relied solely on visualizing the crude to adjusted outcomes, whereas 16 studies used only statistical metrics, such as discrimination and calibration. Finally, 17 studies provided both visualizations and statistical performance metrics (Supplementary Appendix 4, available at https://doi.org/10.1016/j.esmorw.2025.100176).

Twenty-five studies explicitly aimed to develop case-mix adjustment models for benchmarking between hospitals. However, seven of these studies did not describe the details of their models, and six failed to report visual or statistical performance metrics. Internal validation was conducted in 12 of the 25 studies. Seven^13^^,^^14^^,^^27^^,^^40^^,^^59^^,^^64^^,^^71^ of the 25 model development studies provided sufficient detail regarding their models, reported performance metrics, and carried out internal validation, ensuring transparency, validity, and reproducibility (Supplementary Appendix 4, available at https://doi.org/10.1016/j.esmorw.2025.100176).

## Discussion

This scoping review evaluated hospital-based benchmarking of cancer outcomes across Europe. Of 4950 screened articles, 65 met the inclusion criteria, encompassing studies from eight countries, primarily the Netherlands and the United Kingdom. Most studies benchmarked short-term post-operative outcomes, such as 30-day mortality and morbidity. Surprisingly, only 10 studies focused on long-term survival, and just 2 addressed PROs. Case-mix adjustments commonly included demographic variables, comorbidity indices, functional status assessments, and tumor stage, whereas socioeconomic factors were notably underrepresented. More than half of the studies inadequately adjusted for treatment variables within the control of participating hospitals, potentially obscuring true performance differences. Traditional regression models remain the gold standard for case-mix adjustment, with advanced ML methods offering limited performance gains at the expense of transparency. The optimal approach for evaluating case-mix models remains unclear. Visualizing crude to adjusted outcomes enhances clinical interpretability, whereas discrimination and calibration metrics provide statistical validation. Ideally, both approaches should be combined to ensure both clinical relevance and methodological robustness. Furthermore, there is no consensus on how to report case-mix models. Of the 25 studies specifically aimed at developing case-mix models, only 7 provided sufficient detail for reproducibility, carried out internal validation, and presented performance metrics.

The strengths of this scoping review include a rigorous method with two blind reviewers screening and assessing the full text of all the papers. The second main strength is that the team that conducted this review is multinational with extensive experience in benchmarking cancer outcomes in Europe. The main limitation of this review is its geographical focus on Europe. A global scope would have been valuable but was deemed too extensive. Even within Europe, variations in health care data pose challenges, and expanding internationally would introduce greater heterogeneity, potentially compromising validity. However, given Europe’s advanced role in benchmarking research, we expect this focus to yield meaningful insights. A second limitation is the exclusion of gray literature, which could have increased comprehensiveness but also increased the risk of low-quality data leading to misleading conclusions.

Most studies benchmarked short-term outcomes within 30 days post-surgery, likely due to their ease of measurement and potential to drive rapid quality improvements. However, such focus risks misaligning hospital priorities by emphasizing immediate post-operative results over long-term outcomes. Short-term outcomes often reflect isolated care steps, such as surgery, whereas long-term outcomes capture the success of the entire care continuum, promoting cross-disciplinary collaboration. A balanced benchmarking framework should incorporate short-term actionable metrics and long-term outcomes to provide a comprehensive assessment of care quality, ensuring health care systems prioritize outcomes that truly matter to patients. PROs reflect patients’ perspectives on well-being, symptom burden, and functional status, yet they are underutilized in cancer care benchmarking. Only two studies^39^^,^^47^ benchmarked PROs, exposing a significant gap in practice. A reason for this could be that PROs are not yet routinely collected in clinical practice. PROs are inherently influenced by individual, social, and psychological factors, and, as a recent review highlighted,^73^ there is a need for a standardized approach to case-mix adjustment for PROs. Further research is needed to determine whether, how, and which PROs can be used for valid benchmarking. TOs present a promising approach to capture different aspects of quality of care into a single metric. Achieving TO has also been associated with improved long-term survival in several studies,^74^^,^^75^ potentially bridging the gap between short- and long-term outcomes. However, a TO is usually binary in nature, where the outcome is achieved if all included subcomponents are met. This all-or-nothing approach can obscure actionable insights, as underperformance offers no clarity on whether issues stem from surgical technique, perioperative care, or follow-up. Assessing individual TO components may help hospitals identify targeted areas for improvement. Moreover, none of the studies using TOs reported case-mix model validation metrics, such as discrimination or calibration, which are critical for assessing reliability. Standardizing cancer outcome measures across countries is essential for effective international benchmarking. This can be achieved, for example, by aligning with the International Consortium of Health Outcome Measurement sets.^76^

Sixty percent of the studies adjusted their results for treatment-related variables, such as the choice of oncological interventions or surgical techniques. The choice of treatment clearly affects the risk of complications and mortality. It may therefore seem intuitive to adjust for these factors. However, this approach poses challenges in benchmarking. Treatment-related adjustments are often within hospitals’ direct control and adjusting for them may inadvertently mask hospital-level variations that could highlight areas for improvement. Moreover, because clinical guidelines define standard care practices, adjusting for treatment variables may conceal deviations from these recommendations that signal opportunities for enhancing care. A more effective strategy is to adjust for tumor stage or other indicators of disease complexity, reflecting the expected difficulty of treatment without obscuring hospital performance in delivering the most appropriate care.

One significant concern arising from our findings is the low representation of socioeconomic factors in case-mix adjustments. Although socioeconomic status is a well-established determinant of health outcomes, it was often not considered in the included studies. This omission may lead to unfair benchmarking by obscuring the true performance of hospitals serving more disadvantaged populations. Among the 14 studies that included socioeconomic adjustors, 6 did not report sufficient model details to assess their impact. The findings were mixed among the remaining eight studies. Future research should clarify whether including socioeconomic factors enhances the accuracy of case-mix models, thereby supporting more equitable benchmarking.

The choice of case-mix variables is crucial, as these directly impact the interpretation of outcomes across hospitals. We argue that research teams developing these models should adopt an interdisciplinary approach—engaging experienced clinicians to identify clinically relevant factors alongside biostatisticians and epidemiologists with expertise in statistical modeling. This collaboration ensures that the selected variables are both clinically pertinent and methodologically robust. Moreover, transparency in the selection and application of case-mix factors is vital for reproducibility and for fostering trust in benchmarking results. Another key consideration is that outcome indicators are differentially influenced by case-mix. A recent study^77^—published after our review’s cut-off date—reported that the type of breast cancer surgery is the predominant predictor of post-operative complication rates. After stratifying by type of surgery, additional case-mix adjustment did not alter hospital rankings, suggesting a negligible impact of such adjustments in that context. Given that collecting case-mix data is resource intensive, it is imperative to critically evaluate its impact to ensure that the effort yields meaningful improvements in benchmarking.

Our analysis highlights the predominance of traditional regression modeling for case-mix adjustment, reflecting their methodological reliability and widespread acceptance. Classical regression generally performs well when relationships between predictors and outcomes are fairly linear, and the key variables are well understood. However, these models can struggle to capture complex nonlinear relationships and interactions between case-mix factors, which in turn could favor more complex modeling.

LCM offers a novel approach by identifying unobserved patient subgroups with distinct risk profiles. This represents a promising intermediate method with characteristics of both classical regression and advanced ML. LCM could potentially uncover hidden subgroups of risk that classical regression fails to capture, but it is yet to be explored whether this theoretical advantage can translate into real-world improvements in cancer outcome benchmarking, and in which contexts these advantages outweigh the increased model complexity and limited transparency.

Only a small number of studies in our review employed more advanced ML techniques, such as decision trees or gradient boosting. This limited exploration may represent a missed opportunity for innovation, but it must be weighed against important considerations. Advanced ML models generally require large, high-quality datasets to avoid overfitting and achieve reliable performance—a condition not always met in real-world oncology data. The added complexity and ‘black box’ nature of many ML models have rightly raised concerns about interpretability and clinical trust. However, advances in explainable artificial intelligence (AI) techniques^78^ offer promising avenues to enhance transparency and support clinical adoption.

In our review, advanced ML methods showed only modest performance gains compared with classical regression. Notably, most of the included studies developed models on relatively small datasets, which likely limited the potential of ML approaches. One exception was a study using a dataset of 62 000 patients and 103 preoperative variables.^64^ Even in this large, rich dataset, advanced ML techniques—including random forests, gradient boosting, and elastic net regression—provided none to minimal improvements in discrimination over classical logistic regression. This aligns with previous evidence showing that advanced ML techniques often do not outperform logistic regression in clinical prediction modeling.^79^

Ultimately, the choice of statistical approach for case-mix adjustment should be guided by the clinical context. Simple, transparent regression models may suffice—and even excel—when predictor–outcome relationships are well understood or when sample sizes are limited. A pragmatic approach is to consider advanced ML methods when classical regression performs inadequately—such as in cases of poor calibration, low discrimination, or limited clinical plausibility. Scenarios involving complex nonlinear relationships, high-order interactions, or a large number of predictors could theoretically favor ML. However, without clear evidence of added value, simpler and more interpretable models remain the more practical and defensible choice in most benchmarking contexts. A systematic framework for evaluating when ML techniques offer meaningful advantages would help advance methodological rigor in cancer outcome benchmarking.

There is currently no consensus on how to best evaluate case-mix models. In our review, two main strategies emerged: some studies relied on visualizations of hospital outcomes before and after adjustment, whereas others used statistical performance metrics like discrimination and calibration, adapted from prediction modeling. However, it is important to recognize that case-mix modeling serves a different purpose than prediction modeling. While prediction models aim to estimate an individual patient’s risk, case-mix models are used to adjust hospital-level outcomes to account for differences in patient populations. This means the priorities for model assessment are also different. In prediction modeling, discrimination—the ability to distinguish between patients who experience the outcome and those who do not—is often emphasized. In contrast, case-mix models prioritize calibration, ensuring that the model accurately predicts outcomes across different risk groups so that hospitals treating sicker or more complex patients are not unfairly penalized.

Although metrics like discrimination and calibration provide an objective view of model performance, they do not show how case-mix adjustment affects hospital rankings or performance comparisons. Visualizations—such as plots of hospital outcomes before and after adjustment—offer a clearer and more intuitive view of how the model works in practice. A combined approach, using both performance metrics and visualizations, offers the most balanced assessment. It captures both statistical validity and practical impact and helps ensure the adjusted outcomes are both fair and clinically meaningful.

Although the TRIPOD guidelines^80^ have set a benchmark for the transparent reporting of individual prediction models, no analogous guidelines exist for case-mix modeling. In our review only 7 of 25 studies that explicitly aimed to develop case-mix models provided sufficient detail regarding their models for reproducibility, reported performance metrics, and carried out internal validation. In Table 2 we summarize our suggestions for developing case-mix models in a cancer outcome benchmarking context. These suggestions are intended to support more transparent, reproducible, and clinically meaningful benchmarking efforts, particularly in the absence of formal consensus guidelines. The development of dedicated reporting standards for case-mix modeling would facilitate standardized model description, validation, and assessment across studies. This, in turn, would enable more meaningful cross-study comparisons, helping to identify which disease areas and outcome domains are well served by current benchmarking practices and where methodological improvements are still needed. Moreover, standardized practices are essential for advancing international benchmarking, as it remains an open question whether case-mix models can be generalized across health care systems and countries. Furthermore, standardized guidelines would facilitate external validation and model reuse, improve peer review practices, and enable systematic evidence synthesis—critical steps toward establishing benchmarking as a rigorous, generalizable, and collaborative field.

**Table 2:** Recommendations for conducting cancer benchmarking studies

Category	Suggestion
Case-mix factors	
	Select case-mix factors based on clinical expertise and prior knowledge (i.e. hypothesis-driven selection)
	Case-mix variables should be available before treatment (i.e. at the time of diagnosis or treatment planning)
	Avoid including treatment factors in the case-mix model
	Consider adding socioeconomic factors if available and deemed relevant
Statistical approach	
	Transparent regression can be applied in most cancer benchmarking settings
	Consider advanced machine learning methods when traditional regression shows poor performance, especially in settings with complex, high-dimensional, or nonlinear data
Model assessment	
	Clarify that the model is intended for benchmarking purposes—not individual outcome prediction—as this guides how performance metrics like calibration and discrimination should be interpreted, and how the model can be used
	Statistical performance should, at minimum, be assessed by model discrimination and model calibration
	Assess clinical validity using visualizations that show how hospital outcomes shift from crude to adjusted, making the impact of case-mix modeling transparent
	Perform internal validation to mitigate the risk of overfitting
Reporting	
	Clearly report which case-mix factors were considered, which were included in the final model, and provide justification for inclusion/exclusion
	Clearly report model performance and validation results, including any limitations or potential biases

To our knowledge, this is the first scoping review on benchmarking practices in cancer care. Hospital benchmarking of cancer outcomes is essential to improving both quality and equity in cancer care across Europe. However, significant methodological challenges remain. A shift toward standardized, validated benchmarking practices is essential to drive meaningful improvements. Future benchmarking frameworks must integrate validated case-mix models, incorporate standardized outcome sets, especially focusing on long-term outcomes and PROs, and refine adjustment strategies to ensure fair and meaningful comparisons. A consensus on case-mix model assessment and dedicated reporting guidelines, akin to the TRIPOD statement, are urgently needed to ensure transparency and comparability. Addressing these challenges will enable robust international comparisons, promote equity in cancer care, and ensure benchmarking leads to tangible improvements in patient outcomes across Europe. Only through rigorous methodologies, standardized reporting, and international collaboration can hospital benchmarking become a transformative tool for cancer care improvement.

## References

[1] Ettorchi-Tardy A., Levif M., Michel P. (2012). Benchmarking: a method for continuous quality improvement in health. Healthc Policy.

[2] Agency for health care research quality Types of health care quality measures. https://www.ahrq.gov/talkingquality/measures/types.html.

[3] Iezzoni L.I., Mossialos E., Papanicolas I., Smith P.C., Leatherman S. (2010). Performance Measurement for Health System Improvement: Experiences, Challenges and Prospects.

[4] Tricco A.C., Lillie E., Zarin W. (2018). PRISMA extension for scoping reviews (PRISMA-ScR): checklist and explanation. Ann Intern Med.

[5] Wells G., Shea B., O’Connell D. (2014). Newcastle-Ottawa quality assessment scale cohort studies. https://www.ohri.ca/programs/clinical_epidemiology/nosgen.pdf.

[6] Barker T.H., Hasanoff S., Aromataris E. (2025). The revised JBI critical appraisal tool for the assessment of risk of bias for cohort studies. JBI Evid Synth.

[7] Institute J.B. (2024). JBI manual for evidence synthesis – scoping reviews. https://jbi-global-wiki.refined.site/space/MANUAL/355863071/10.3.7.3+Data+extraction.

[8] Algera M.D., Baldewpersad Tewarie N.M.S., van Driel W.J. (2023). Participants of the Dutch Gynecological Oncology Audit Collaborator Group. Case-mix adjustment to compare hospital performances regarding complications after cytoreductive surgery for ovarian cancer: a nationwide population-based study. Int J Gynecol Cancer.

[9] Algera M.D., Slangen B.F.M., van Driel W.J., Wouters M.W.J.M., Kruitwagen R.F.P.M., Participants of the Dutch Gynecological Oncology Audit Collaborator Group (2023). Textbook outcome as a composite outcome measure to compare hospital performances regarding cytoreductive surgery for ovarian cancer: a nationwide population-based study. Gynecol Oncol.

[10] Aravani A., Samy E.F., Thomas J.D., Quirke P., Morris E.J.A., Finan P.J. (2016). A retrospective observational study of length of stay in hospital after colorectal cancer surgery in England (1998-2010). Medicine (Baltimore).

[11] Beck N., Hoeijmakers F., van der Willik E.M. (2018). National comparison of hospital performances in lung cancer surgery: the role of case mix adjustment. Ann Thorac Surg.

[12] Bernard A., Cottenet J., Pagès P.B., Quantin C. (2023). Mortality and failure-to-rescue major complication trends after lung cancer surgery between 2005 and 2020: a nationwide population-based study. BMJ Open.

[13] Bernard A., Rivera C., Pages P.B., Falcoz P.E., Vicaut E., Dahan M. (2011). Risk model of in-hospital mortality after pulmonary resection for cancer: a national database of the French Society of Thoracic and Cardiovascular Surgery (Epithor). J Thorac Cardiovasc Surg.

[14] Blake H.A., Sharples L.D., Boyle J.M. (2024). Improving risk models for patients having emergency bowel cancer surgery using linked electronic health records: a national cohort study. Int J Surg.

[15] Boyle J.M., van der Meulen J., Kuryba A. (2023). Measuring variation in the quality of systemic anti-cancer therapy delivery across hospitals: a national population-based evaluation. Eur J Cancer.

[16] Burnell M., Iyer R., Gentry-Maharaj A. (2016). Benchmarking of surgical complications in gynaecological oncology: prospective multicentre study. BJOG.

[17] Busweiler L.A.D., Schouwenburg M.G., van Berge Henegouwen M.I. (2017). Textbook outcome as a composite measure in oesophagogastric cancer surgery. Br J Surg.

[18] D’Journo X.B., Berbis J., Jougon J. (2017). External validation of a risk score in the prediction of the mortality after esophagectomy for cancer. Dis Esophagus.

[19] Damhuis R., Coonar A., Plaisier P. (2006). A case-mix model for monitoring of postoperative mortality after surgery for lung cancer. Lung Cancer.

[20] Damhuis R.A., Maat A.P., Plaisier P.W. (2015). Performance indicators for lung cancer surgery in the Netherlands. Eur J Cardiothorac Surg.

[21] Das N., Talaat A.S., Naik R. (2006). Risk adjusted surgical audit in gynaecological oncology: P-POSSUM does not predict outcome. Eur J Surg Oncol.

[22] de Graaff M.R., Elfrink A.K.E., Buis C.I. (2022). Defining Textbook Outcome in liver surgery and assessment of hospital variation: a nationwide population-based study. Eur J Surg Oncol.

[23] de Graaff M.R., Klaase J.M., van Dam R.M. (2023). Survival of patients with colorectal liver metastases treated with and without preoperative chemotherapy: nationwide propensity score-matched study. Eur J Surg Oncol.

[24] Elfrink A.K.E., Kok N.F.M., Swijnenburg R.J. (2022). Nationwide oncological networks for resection of colorectal liver metastases in the Netherlands: differences and postoperative outcomes. Eur J Surg Oncol.

[25] Elfrink A.K.E., Olthof P.B., Swijnenburg R.J. (2021). Factors associated with failure to rescue after liver resection and impact on hospital variation: a nationwide population-based study. HPB (Oxford).

[26] Elfrink A.K.E., van Zwet E.W., Swijnenburg R.J. (2021). Case-mix adjustment to compare nationwide hospital performances after resection of colorectal liver metastases. Eur J Surg Oncol.

[27] Fischer C., Lingsma H., Hardwick R., Cromwell D.A., Steyerberg E., Groene O. (2016). Risk adjustment models for short-term outcomes after surgical resection for oesophagogastric cancer. Br J Surg.

[28] Fischer C., Lingsma H.F., van Leersum N., Tollenaar R.A., Wouters M.W., Steyerberg E.W. (2015). Comparing colon cancer outcomes: the impact of low hospital case volume and case-mix adjustment. Eur J Surg Oncol.

[29] Gildea C., Nordin A., Hirschowitz L., Poole J. (2016). Thirty-day postoperative mortality for endometrial carcinoma in England: a population-based study. BJOG.

[30] Gilthorpe M.S., Harrison W.J., Downing A., Forman D., West R.M. (2011). Multilevel latent class casemix modelling: a novel approach to accommodate patient casemix. BMC Health Serv Res.

[31] Govaert J.A., van Dijk W.A., Fiocco M. (2016). Nationwide outcomes measurement in colorectal cancer surgery: improving quality and reducing costs. J Am Coll Surg.

[32] Gray E., Figueroa J.D., Oikonomidou O. (2021). Variation in chemotherapy prescribing rates and mortality in early breast cancer over two decades: a national data linkage study. ESMO Open.

[33] Greijdanus N.G., van Erning F.N., van Workum F., Tanis P.J., de Wilt J.H.W., Vissers P.A.J. (2024). Variation in hospital performances after colorectal cancer surgery: a case-mix adjusted Dutch population based study. Eur J Surg Oncol.

[34] Henneman D., Snijders H.S., Fiocco M. (2013). Hospital variation in failure to rescue after colorectal cancer surgery: results of the Dutch Surgical Colorectal Audit. Ann Surg Oncol.

[35] Henneman D., van Bommel A.C., Snijders A. (2014). Ranking and rankability of hospital postoperative mortality rates in colorectal cancer surgery. Ann Surg.

[36] Jack R.H., Gulliford M.C., Ferguson J., Moller H. (2003). Geographical inequalities in lung cancer management and survival in South East England: evidence of variation in access to oncology services?. Br J Cancer.

[37] Kolfschoten N.E., Kievit J., Gooiker G.A. (2013). Focusing on desired outcomes of care after colon cancer resections; hospital variations in ‘textbook outcome’. Eur J Surg Oncol.

[38] Kolfschoten N.E., Marang van de Mheen P.J., Gooiker G.A. (2011). Variation in case-mix between hospitals treating colorectal cancer patients in the Netherlands. Eur J Surg Oncol.

[39] Kowalski C., Sibert N.T., Breidenbach C. (2022). Outcome quality after colorectal cancer resection in Certified Colorectal Cancer Centers-patient-reported and short-term clinical outcomes. Dtsch Arztebl Int.

[40] Matthes-Martin S., Potschger U., Bergmann K. (2008). Risk-adjusted outcome measurement in pediatric allogeneic stem cell transplantation. Biol Blood Marrow Transplant.

[41] McArdle C.S., Hole D.J. (2002). Outcome following surgery for colorectal cancer: analysis by hospital after adjustment for case-mix and deprivation. Br J Cancer.

[42] Morris E.J.A., Taylor E.F., Thomas J.D. (2011). Thirty-day postoperative mortality after colorectal cancer surgery in England. Gut.

[43] Myrdal G., Lamberg K., Lambe M., Ståhle E., Wagenius G., Holmberg L. (2009). Regional differences in treatment and outcome in non-small cell lung cancer: a population-based study (Sweden). Lung Cancer.

[44] Nickelsen T.N., Jørgensen T., Kronborg O. (2005). Thirty-day mortality after surgery for colorectal cancer in Denmark. Colorectal Dis.

[45] Nilssen Y., Brustugun O.T., Fjellbirkeland L. (2024). Small cell lung cancer in norway: patterns of care by health region and survival trends. Clin Lung Cancer.

[46] Salet N., Stangenberger V.A., Bremmer R.H., Eijkenaar F. (2023). Between-hospital and between-physician variation in outcomes and costs in high- and low-complex surgery: a nationwide multilevel analysis. Value Health.

[47] Sibert N.T., Pfaff H., Breidenbach C. (2022). Variation across operating sites in urinary and sexual outcomes after radical prostatectomy in localized and locally advanced prostate cancer. World J Urol.

[48] Skyrud K.D., Bray F., Eriksen M.T., Nilssen Y., Møller B. (2016). Regional variations in cancer survival: impact of tumour stage, socioeconomic status, comorbidity and type of treatment in Norway. Int J Cancer.

[49] Snijders H.S., Henneman D., van Leersum N.L. (2013). Anastomotic leakage as an outcome measure for quality of colorectal cancer surgery. BMJ Qual Saf.

[50] Talsma A.K., Lingsma H.F., Steyerberg E.W., Wijnhoven B.P., Van Lanschot J.J. (2014). The 30-day versus in-hospital and 90-day mortality after esophagectomy as indicators for quality of care. Ann Surg.

[51] Talsma A.K., Reedijk A.M., Damhuis R.A., Westenend P.J., Vles W.J. (2011). Re-resection rates after breast-conserving surgery as a performance indicator: introduction of a case-mix model to allow comparison between Dutch hospitals. Eur J Surg Oncol.

[52] Ten Berge M.G., Beck N., Steup W.H. (2021). Textbook outcome as a composite outcome measure in non-small-cell lung cancer surgery. Eur J Cardiothorac Surg.

[53] Thurell J., Manouchehri N., Fredriksson I. (2023). Risk-adjusted benchmarking of long-term overall survival in patients with HER2-positive early-stage Breast cancer: a Swedish retrospective cohort study. Breast (Edinburgh, Scotland).

[54] Tighe D., Fabris F., Freitas A. (2021). Machine learning methods applied to audit of surgical margins after curative surgery for head and neck cancer. Br J Oral Maxillofac Surg.

[55] Tighe D., Kwok A., Putcha V., McGurk M. (2014). Identification of appropriate outcome indices in head and neck cancer and factors influencing them. Int J Oral Maxillofac Surg.

[56] Tighe D., Lewis-Morris T., Freitas A. (2019). Machine learning methods applied to audit of surgical outcomes after treatment for cancer of the head and neck. Br J Oral Maxillofac Surg.

[57] Tighe D., McMahon J., Schilling C., Ho M., Provost S., Freitas A. (2022). Machine learning methods applied to risk adjustment of cumulative sum chart methodology to audit free flap outcomes after head and neck surgery. Br J Oral Maxillofacial Surg.

[58] Tighe D., Sassoon I., Hills A., Quadros R. (2019). Case-mix adjustment in audit of length of hospital stay in patients operated on for cancer of the head and neck. Br J Oral Maxillofac Surg.

[59] Tighe D., Sassoon I., Kwok A., McGurk M. (2014). Is benchmarking possible in audit of early outcomes after operations for head and neck cancer?. Br J Oral Maxillofac Surg.

[60] Tighe D., Sassoon I., McGurk M. (2017). Validating a benchmarking tool for audit of early outcomes after operations for head and neck cancer. Ann R Coll Surg Engl.

[61] Tighe D., Tekeli K., Gouk T. (2023). Machine learning methods applied to audit of surgical margins after curative surgery for facial (non-melanoma) skin cancer. Br J Oral Maxillofac Surg.

[62] Tighe D., Thomas A.J., Hills A., Quadros R. (2019). Validating a risk stratification tool for audit of early outcome after operations for squamous cell carcinoma of the head and neck. Br J Oral Maxillofac Surg.

[63] Tighe D.F., Thomas A.J., Sassoon I., Kinsman R., McGurk M. (2017). Developing a risk stratification tool for audit of outcome after surgery for head and neck squamous cell carcinoma. Head Neck.

[64] van den Bosch T., Warps A.K., de Nerée Tot Babberich M.P.M. (2021). Predictors of 30-day mortality among Dutch patients undergoing colorectal cancer surgery, 2011-2016. JAMA Netw Open.

[65] van der Heiden-van der Loo M., de Munck L., Visser O. (2012). Variation between hospitals in surgical margins after first breast-conserving surgery in the Netherlands. Breast Cancer Res Treat.

[66] Voeten D.M., van der Werf L.R., van Sandick J.W., van Hillegersberg R., van Berge Henegouwen M.I. (2021). Length of hospital stay after uncomplicated esophagectomy. Hospital variation shows room for nationwide improvement. Surg Endosc.

[67] Voeten D.M., Van Der Werf L.R., Wijnhoven B.P.L., Van Hillegersberg R., Van Berge Henegouwen M.I. (2020). Failure to cure in patients undergoing surgery for esophageal carcinoma: hospital of surgery influences prospects for cure: a nation-wide cohort study. Ann Surg.

[68] Vos E.L., Koppert L.B., Jager A., Vrancken Peeters M., Siesling S., Lingsma H.F. (2020). From multiple quality indicators of breast cancer care toward hospital variation of a summary measure. Value Health.

[69] Vos E.L., Lingsma H.F., Jager A. (2020). Effect of case-mix and random variation on breast cancer care quality indicators and their rankability. Value Health.

[70] Wahba A.J., Phillips N., Mathew R.K., Hutchinson P.J., Helmy A., Cromwell D.A. (2023). Benchmarking short-term postoperative mortality across neurosurgery units: is hospital administrative data good enough for risk-adjustment?. Acta Neurochir (Wien).

[71] Walker K., Finan P.J., van der Meulen J.H. (2015). Model for risk adjustment of postoperative mortality in patients with colorectal cancer. Br J Surg.

[72] Warps A.K., Detering R., Tollenaar R., Tanis P.J., Dekker J.W.T. (2021). Textbook outcome after rectal cancer surgery as a composite measure for quality of care: a population-based study. Eur J Surg Oncol.

[73] Sibert N.T., Pfaff H., Breidenbach C., Wesselmann S., Kowalski C. (2021). Different approaches for case-mix adjustment of patient-reported outcomes to compare health care providers-methodological results of a systematic review. Cancers (Basel).

[74] Dal Cero M., Roman M., Grande L. (2022). Textbook outcome and survival after gastric cancer resection with curative intent: a population-based analysis. Eur J Surg Oncol.

[75] Xu S.J., Lin L.Q., Chen C. (2022). Textbook outcome after minimally invasive esophagectomy is an important prognostic indicator for predicting long-term oncological outcomes with locally advanced esophageal squamous cell carcinoma. Ann Transl Med.

[76] ICHOM Patient-centered outcome measures. https://www.ichom.org/patient-centered-outcome-measures/.

[77] Verheul E.M., van Klaveren D., Lingsma H.F. (2024). High-impact complications after breast cancer surgery in the Dutch national quality registry: evaluating case-mix adjustment for hospital comparisons. BJS Open.

[78] Gunning D., Aha D. (2019). DARPA’s explainable artificial intelligence (XAI) program. AI Magazine.

[79] Christodoulou E., Ma J., Collins G.S., Steyerberg E.W., Verbakel J.Y., Van Calster B. (2019). A systematic review shows no performance benefit of machine learning over logistic regression for clinical prediction models. J Clin Epidemiol.

[80] Collins G.S., Reitsma J.B., Altman D.G., Moons K.G. (2015). Transparent reporting of a multivariable prediction model for individual prognosis or diagnosis (TRIPOD): the TRIPOD statement. Br Med J.

