# Supplementary material for: Benchmarking cancer outcomes in Europe: a scoping review of methodologies and case-mix adjustments
Source: ESMO Real World Data Digit Oncol. 2025 Sep 3;9:100176. doi: 10.1016/j.esmorw.2025.100176 (PMC12836561; doi:10.1016/j.esmorw.2025.100176)
Supplement: Supplementary Material [file mmc1.docx]

**Appendix 1 –** Documentation of search strings

Documentation of search strategies

University Library search consultation group

Date: May 2024

Topic/research question: risk adjustment and benchmarking in cancer care

Name of researcher(s): XXX

Librarian(s): XXX Peer reviewed by XXX

Databases:

1. Medline (Ovid)
2. Embase (embase.com)
3. Web of Science Core Collection (Clarivate)

Total number of hits:

- Before deduplication: 4771
- After deduplication: 2571

Text that can be used in the Methods-section:

Search strategy

A literature search was performed in the following databases: Medline, Embase, and Web of Science. The last search was conducted 2024-05-30.

After the original search was performed on 30 May 2024, the search was last updated on 11 November 2024 by rerunning the searches and deduplicating against the previous results using Covidence (1).

The search strategy was developed in Medline (Ovid) in collaboration with librarians at the For each search concept Medical Subject Headings (MeSH-terms) and free text terms were identified. The search was then translated, in part using Polyglot Search Translator (2), into the other databases.

No language restriction was applied-

Databases were searched from inception.

The strategies were peer reviewed by another librarian prior to execution.

De-duplication was done using the method described by Bramer et al (3). One final, extra step was added to compare DOIs.

A snow-ball search was applied to check references and citations of eligible studies from the database searches using spidercite (4).

The full search strategies for all databases are available in the appendix.

References

1. Covidence systematic review software, Veritas Health Innovation, Melbourne, Australia. Available at www.covidence.org.

2. Clark JM, Sanders S, Carter M, Honeyman D, Cleo G, Auld Y, Booth D, Condron P, Dalais C, Bateup S, Linthwaite B, May N, Munn J, Ramsay L, Rickett K, Rutter C, Smith A, Sondergeld P, Wallin M, Jones M, Beller E. (2020) Improving the translation of search strategies using the Polyglot Search Translator: a randomized controlled trial. *Journal of the Medical Library Association: JMLA*. 108(2):195-207. doi: 10.5195/jmla.2020.834.

3. Bramer, W. M., Giustini, D., de Jonge, G. B., Holland, L., & Bekhuis, T. (2016). De-duplication of database search results for systematic reviews in EndNote. *Journal of the Medical Library Association: JMLA*, 104(3), 240-243. doi: 10.3163/1536-5050.104.3.014

4. Institute for Evidence-based Healthcare. SR-accelerator systematic review accelerator published 2021. Accessed November 12, 2024. https://sr-accelerator.com/#/help/spidercite.


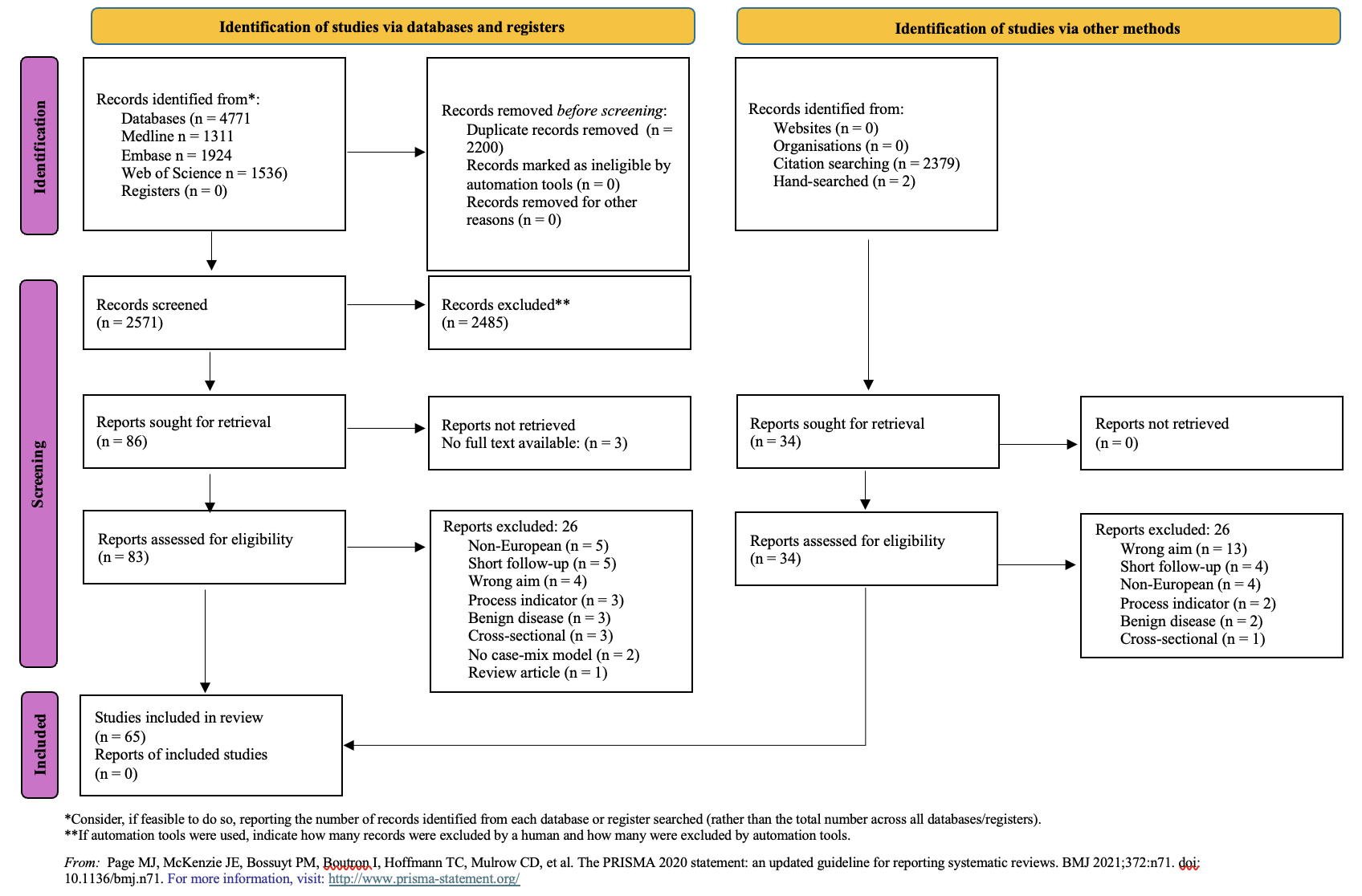


1. Medline

| Interface: Ovid MEDLINE(R) ALL  Date of Search: 2024-11-11  Number of hits: 1311  Comment: In Ovid, two or more words are automatically searched as phrases; i.e. no quotation marks are needed | Field labels   - exp/ = exploded MeSH term - / = non exploded MeSH term - .ti,ab,kf. = title, abstract and author keywords - adjx = within x words, regardless of order - * = truncation of word for alternate endings |
| --- | --- |
| Ovid MEDLINE(R) ALL <1946 to November 08, 2024>   \| 1 \| Benchmarking/ \| 19163 \| \| --- \| --- \| --- \| \| 2 \| (Benchmark* or bench-mark* or best practice* or metric*).ti,ab,kf. \| 236656 \| \| 3 \| Quality Indicators, Health Care/ \| 17854 \| \| 4 \| ((quality or performance) adj2 (assessment* or assurance or indicator* or measur*)).ti,ab,kf. \| 158400 \| \| 5 \| Outcome Assessment, Health Care/ \| 83853 \| \| 6 \| Standard of Care/ \| 4097 \| \| 7 \| (outcome adj2 (assessment* or indicator* or measure*)).ti,ab,kf. \| 323944 \| \| 8 \| Medical Audit/ \| 17538 \| \| 9 \| Clinical Audit/ \| 2102 \| \| 10 \| ((clinical or medical) adj1 audit*).ti,ab,kf. \| 4745 \| \| 11 \| ((countr* or nation* or healthcare or health or hospital* or regional*) adj5 (compar* or differenc* or disparit* or inequit* or inequalit* or variation*)).ti,ab,kf. \| 289911 \| \| 12 \| Quality assurance, Health Care/ \| 57153 \| \| 13 \| Healthcare Disparities/ \| 24921 \| \| 14 \| (care adj2 standard*).ti,ab,kf. \| 82162 \| \| 15 \| textbook outcome*.ti,ab,kf. \| 368 \| \| 16 \| or/1-15 \| 1174947 \| \| 17 \| exp Neoplasms/ \| 4039579 \| \| 18 \| (acanthoma* or adamantinoma* or adenoameloblastoma* or adenocarcinoma* or adenofibroma* or adenolipoma* or adenolymphoma* or adenoma* or adenomyoepithelioma* or adenomyoma* or adenosarcoma* or aesthesioneuroblastoma* or ameloblastoma* or androblastoma* or angioblastoma* or angiofibroma* or angiofibrosarcoma* or angioleiomyoma* or angiolipoma* or angiomyolipoma* or angiomyoma* or angiomyxoma* or angiosarcoma* or arrhenoblastoma* or astroblastoma* or astrocytoma* or astroglioma* or blastoma* or cancer* or carcinoid or carcinoma* or carcinosarcoma* or cholangio* or cholangiocarcinoma* or cholangiosarcoma* or chondro* or chondroblastoma* or chondrosarcoma* or chorio* or chorioadenoma* or choriocarcinoma* or chorioepithelioma* or chorionepithelioma* or choristoma* or comedocarcinoma* or cystadenocarcinoma* or cystadenofibroma* or cystadenoma* or cystosarcoma* or dermatofibroma* or dermatofibrosarcoma* or elastofibroma* or enchondroma* or endothelioma* or ependymoblastoma* or ependymoma* or epithelioma* or erythroleukemia* or esthesioneuroblastoma* or esthesioneuroepithelioma* or fibroadenoma* or fibroadenosarcoma* or fibrochondrosarcoma* or fibroepithelioma* or fibroid* or fibrolipoma* or fibroliposarcoma* or fibroma* or fibromatosis or fibromyoma* or fibromyxolipoma* or fibromyxoma* or fibrosarcoma* or fibroxanthosarcoma* or ganglioblastoma* or gangliocytoma* or ganglioglioma* or ganglioneuroblastoma* or ganglioneurofibroma* or ganglioneuroma* or gastrinoma* or germinoma* or glioblastoma* or gliofibroma* or glioma* or glioneuroma* or gliosarcoma* or glomangiomyoma* or glomangiosarcoma* or glucagonoma or gonadoblastoma* or gonocytoma* or germinoblastoma* or gynandroblastoma* or haemangioblastoma* or haemangioma* or haemangiopericytoma* or haemangiosarcoma* or hamartoma* or hemangioblastoma* or hemangioendotheliosarcoma* or hemangiopericytoma* or hemangiosarcoma* or hepatoblastoma* or hepatocarcinoma* or hepatocholangiocarcinoma* or hepatoma* or hidradenoma* or histiocytoma* or hodgkin* or hydradenoma* or immunocytoma* or leiomyoblastoma* or leiomyofibroma* or leiomyoma* or leiomyosarcoma* or leucocythaemia or leucocythemia or leukemi* or lipoadenoma* or lipoblastoma* or lipoma* or liposarcoma* or luteoma* or lymphangiomyoma* or lymphangiosarcoma* or lymphoepithelioma* or lymphoma* or lymphosarcoma or malignancy or malignant or masculinovoblastoma* or mastocytoma* or medulloblastoma* or medullocytoma* or medulloepithelioma* or medullomyoblastoma* or melanoameloblastoma* or melanocytoma* or melanom* or meningioblastoma* or mesenchymoma* or mesothelioma* or metastasis or microglioma* or myelolipoma* or myeloma* or myoblastoma* or myoepithelioma* or myofibroblastoma* or myofibroma* or myofibrosarcoma* or myolipoma* or myoma* or myosarcoma* or myxofibroma* or myxolipoma* or myxoliposarcoma* or myxoma* or neoplas* or nephroblastoma* or neurilemmoma* or neurilemoma* or neuroblastoma* or neurocytoma* or neuroepithelioma* or neurofibroma* or neurofibrosarcoma* or neurolipocytoma* or neuroma* or neuroma* or odontoameloblastoma* or oligoastrocytoma* or oligodendroglioma* or oncocytoma* or orchioblastoma* or osteoblastoma* or osteochondroma* or osteochondrosarcoma* or osteoclastoma* or osteofibrosarcoma* or osteosarcoma* or pancreatoblastoma* or paraganglioma* or phaeochromocytoma* or pheochromoblastoma* or pheochromocytoma* or pinealblastoma* or pinealoblastoma* or pineoblastoma* or pineocytoma* or plasmacytoma* or pneumoblastoma* or pneumocytoma* or porocarcinoma* or preleukemia* or prolactinoma* or reticulohistiocytoma* or reticulolymphosarcoma or retinoblastoma* or rhabdomyoma* or rhabdomyosarcoma* or rhabdosarcoma* or sarcoma* or seminoma* or spermatocytoma* or spiradenoma* or spongioblastoma* or subependymoma* or syringadenoma* or syringocystadenoma* or syringoma* or teratocarcinoma* or teratoma* or thecoma* or thymolipoma* or thymoma* or trichilemmoma* or trichoadenoma* or trichoblastoma* or trichoepithelioma* or tricholemmoma* or tumor* or tumour* or xanthoastrocytoma* or xanthofibroma* or xanthosarcoma*).ti,ab,kf. \| 5078539 \| \| 19 \| Oncology Service, Hospital/ \| 1525 \| \| 20 \| oncology.ti,ab,kf. \| 159255 \| \| 21 \| or/17-20 \| 5738449 \| \| 22 \| Risk Adjustment/ \| 4173 \| \| 23 \| ((risk or case mix or case-mix or casemix) adj3 adjust*).ti,ab,kf. \| 47214 \| \| 24 \| risk-adjust*.ti,ab,kf. \| 13041 \| \| 25 \| or/22-24 \| 49416 \| \| 26 \| 16 and 21 and 25 \| 1311 \| | |

2. Embase

| Interface: embase.com  Date of Search: 2024-11-11  Number of hits: 1924  Comment: Emtree is the controlled vocabulary in Embase | Field labels   - /exp = exploded Emtree term - /de = non exploded Emtree term - ti,ab,kw = title, abstract and author keywords - NEAR/x = within x words, regardless of order - * = truncation of word for alternate endings |
| --- | --- |
| \| No. \| Query \| Results \| \| --- \| --- \| --- \| \| #25 \| #23 NOT #24 \| 1924 \| \| #24 \| #13 AND #18 AND #22 AND ([conference abstract]/lim OR [conference paper]/lim OR [conference review]/lim) \| 1175 \| \| #23 \| #13 AND #18 AND #22 \| 3099 \| \| #22 \| #19 OR #20 OR #21 \| 70216 \| \| #21 \| 'risk adjust*':ti,ab,kw \| 18614 \| \| #20 \| ((risk OR 'case mix' OR 'case mix' OR casemix) NEAR/3 adjust*):ti,ab,kw \| 66968 \| \| #19 \| 'case mix'/de \| 4747 \| \| #18 \| #14 OR #15 OR #16 OR #17 \| 7897232 \| \| #17 \| oncology:ti,ab,kw \| 295174 \| \| #16 \| 'oncology'/exp \| 188379 \| \| #15 \| acanthoma*:ti,ab,kw OR adamantinoma*:ti,ab,kw OR adenoameloblastoma*:ti,ab,kw OR adenocarcinoma*:ti,ab,kw OR adenofibroma*:ti,ab,kw OR adenolipoma*:ti,ab,kw OR adenolymphoma*:ti,ab,kw OR adenoma*:ti,ab,kw OR adenomyoepithelioma*:ti,ab,kw OR adenomyoma*:ti,ab,kw OR adenosarcoma*:ti,ab,kw OR aesthesioneuroblastoma*:ti,ab,kw OR ameloblastoma*:ti,ab,kw OR androblastoma*:ti,ab,kw OR angioblastoma*:ti,ab,kw OR angiofibroma*:ti,ab,kw OR angiofibrosarcoma*:ti,ab,kw OR angioleiomyoma*:ti,ab,kw OR angiolipoma*:ti,ab,kw OR angiomyolipoma*:ti,ab,kw OR angiomyoma*:ti,ab,kw OR angiomyxoma*:ti,ab,kw OR angiosarcoma*:ti,ab,kw OR arrhenoblastoma*:ti,ab,kw OR astroblastoma*:ti,ab,kw OR astrocytoma*:ti,ab,kw OR astroglioma*:ti,ab,kw OR blastoma*:ti,ab,kw OR cancer*:ti,ab,kw OR carcinoid:ti,ab,kw OR carcinoma*:ti,ab,kw OR carcinosarcoma*:ti,ab,kw OR cholangiocarcinoma*:ti,ab,kw OR cholangiosarcoma*:ti,ab,kw OR chondroblastoma*:ti,ab,kw OR chondrosarcoma*:ti,ab,kw OR chorio*:ti,ab,kw OR chorioadenoma*:ti,ab,kw OR choriocarcinoma*:ti,ab,kw OR chorioepithelioma*:ti,ab,kw OR chorionepithelioma*:ti,ab,kw OR choristoma*:ti,ab,kw OR comedocarcinoma*:ti,ab,kw OR cystadenocarcinoma*:ti,ab,kw OR cystadenofibroma*:ti,ab,kw OR cystadenoma*:ti,ab,kw OR cystosarcoma*:ti,ab,kw OR dermatofibroma*:ti,ab,kw OR dermatofibrosarcoma*:ti,ab,kw OR elastofibroma*:ti,ab,kw OR enchondroma*:ti,ab,kw OR endothelioma*:ti,ab,kw OR ependymoblastoma*:ti,ab,kw OR ependymoma*:ti,ab,kw OR epithelioma*:ti,ab,kw OR erythroleukemia*:ti,ab,kw OR esthesioneuroblastoma*:ti,ab,kw OR esthesioneuroepithelioma*:ti,ab,kw OR fibroadenoma*:ti,ab,kw OR fibroadenosarcoma*:ti,ab,kw OR fibrochondrosarcoma*:ti,ab,kw OR fibroepithelioma*:ti,ab,kw OR fibroid*:ti,ab,kw OR fibrolipoma*:ti,ab,kw OR fibroliposarcoma*:ti,ab,kw OR fibromatosis:ti,ab,kw OR fibromyoma*:ti,ab,kw OR fibromyxolipoma*:ti,ab,kw OR fibromyxoma*:ti,ab,kw OR fibrosarcoma*:ti,ab,kw OR fibroxanthosarcoma*:ti,ab,kw OR ganglioblastoma*:ti,ab,kw OR gangliocytoma*:ti,ab,kw OR ganglioglioma*:ti,ab,kw OR ganglioneuroblastoma*:ti,ab,kw OR ganglioneurofibroma*:ti,ab,kw OR ganglioneuroma*:ti,ab,kw OR gastrinoma*:ti,ab,kw OR germinoma*:ti,ab,kw OR glioblastoma*:ti,ab,kw OR gliofibroma*:ti,ab,kw OR glioma*:ti,ab,kw OR glioneuroma*:ti,ab,kw OR gliosarcoma*:ti,ab,kw OR glomangiomyoma*:ti,ab,kw OR glomangiosarcoma*:ti,ab,kw OR glucagonoma:ti,ab,kw OR gonadoblastoma*:ti,ab,kw OR gonocytoma*:ti,ab,kw OR germinoblastoma*:ti,ab,kw OR gynandroblastoma*:ti,ab,kw OR h$emangioblastoma*:ti,ab,kw OR h$emangioma*:ti,ab,kw OR h$emangiopericytoma*:ti,ab,kw OR h$emangiosarcoma*:ti,ab,kw OR h$emartoma*:ti,ab,kw OR h$emangioendotheliosarcoma*:ti,ab,kw OR h$epatoblastoma*:ti,ab,kw OR h$epatocarcinoma*:ti,ab,kw OR h$epatocholangiocarcinoma*:ti,ab,kw OR h$epatoma*:ti,ab,kw OR hidradenoma*:ti,ab,kw OR histiocytoma*:ti,ab,kw OR hodgkin*:ti,ab,kw OR hydradenoma*:ti,ab,kw OR immunocytoma*:ti,ab,kw OR leiomyoblastoma*:ti,ab,kw OR leiomyofibroma*:ti,ab,kw OR leiomyoma*:ti,ab,kw OR leiomyosarcoma*:ti,ab,kw OR leucocythaemia:ti,ab,kw OR leucocythemia:ti,ab,kw OR leukemi*:ti,ab,kw OR lipoadenoma*:ti,ab,kw OR lipoblastoma*:ti,ab,kw OR lipoma*:ti,ab,kw OR liposarcoma*:ti,ab,kw OR luteoma*:ti,ab,kw OR lymphangiomyoma*:ti,ab,kw OR lymphangiosarcoma*:ti,ab,kw OR lymphoepithelioma*:ti,ab,kw OR lymphoma*:ti,ab,kw OR lymphosarcoma:ti,ab,kw OR malignanc*:ti,ab,kw OR malignant:ti,ab,kw OR masculinovoblastoma*:ti,ab,kw OR mastocytoma*:ti,ab,kw OR medulloblastoma*:ti,ab,kw OR medullocytoma*:ti,ab,kw OR medulloepithelioma*:ti,ab,kw OR medullomyoblastoma*:ti,ab,kw OR melanoameloblastoma*:ti,ab,kw OR melanocytoma*:ti,ab,kw OR melanom*:ti,ab,kw OR meningioblastoma*:ti,ab,kw OR mesenchymoma*:ti,ab,kw OR mesothelioma*:ti,ab,kw OR metastasi*:ti,ab,kw OR microglioma*:ti,ab,kw OR myelolipoma*:ti,ab,kw OR myeloma*:ti,ab,kw OR myoblastoma*:ti,ab,kw OR myoepithelioma*:ti,ab,kw OR myofibroblastoma*:ti,ab,kw OR myofibroma*:ti,ab,kw OR myofibrosarcoma*:ti,ab,kw OR myolipoma*:ti,ab,kw OR myoma*:ti,ab,kw OR myosarcoma*:ti,ab,kw OR myxofibroma*:ti,ab,kw OR myxolipoma*:ti,ab,kw OR myxoliposarcoma*:ti,ab,kw OR myxoma*:ti,ab,kw OR neoplas*:ti,ab,kw OR nephroblastoma*:ti,ab,kw OR neurilemmoma*:ti,ab,kw OR neurilemoma*:ti,ab,kw OR neuroblastoma*:ti,ab,kw OR neurocytoma*:ti,ab,kw OR neuroepithelioma*:ti,ab,kw OR neurofibroma*:ti,ab,kw OR neurofibrosarcoma*:ti,ab,kw OR neurolipocytoma*:ti,ab,kw OR neuroma*:ti,ab,kw OR odontoameloblastoma*:ti,ab,kw OR oligoastrocytoma*:ti,ab,kw OR oligodendroglioma*:ti,ab,kw OR oncocytoma*:ti,ab,kw OR orchioblastoma*:ti,ab,kw OR osteoblastoma*:ti,ab,kw OR osteochondroma*:ti,ab,kw OR osteochondrosarcoma*:ti,ab,kw OR osteoclastoma*:ti,ab,kw OR osteofibrosarcoma*:ti,ab,kw OR osteosarcoma*:ti,ab,kw OR pancreatoblastoma*:ti,ab,kw OR paraganglioma*:ti,ab,kw OR phaeochromocytoma*:ti,ab,kw OR pheochromoblastoma*:ti,ab,kw OR pheochromocytoma*:ti,ab,kw OR pinealblastoma*:ti,ab,kw OR pinealoblastoma*:ti,ab,kw OR pineoblastoma*:ti,ab,kw OR pineocytoma*:ti,ab,kw OR plasmacytoma*:ti,ab,kw OR pneumoblastoma*:ti,ab,kw OR pneumocytoma*:ti,ab,kw OR porocarcinoma*:ti,ab,kw OR preleukemia*:ti,ab,kw OR prolactinoma*:ti,ab,kw OR reticulohistiocytoma*:ti,ab,kw OR reticulolymphosarcoma:ti,ab,kw OR retinoblastoma*:ti,ab,kw OR rhabdomyoma*:ti,ab,kw OR rhabdomyosarcoma*:ti,ab,kw OR rhabdosarcoma*:ti,ab,kw OR sarcoma*:ti,ab,kw OR seminoma*:ti,ab,kw OR spermatocytoma*:ti,ab,kw OR spiradenoma*:ti,ab,kw OR spongioblastoma*:ti,ab,kw OR subependymoma*:ti,ab,kw OR syringadenoma*:ti,ab,kw OR syringocystadenoma*:ti,ab,kw OR syringoma*:ti,ab,kw OR teratocarcinoma*:ti,ab,kw OR teratoma*:ti,ab,kw OR thecoma*:ti,ab,kw OR thymolipoma*:ti,ab,kw OR thymoma*:ti,ab,kw OR trichilemmoma*:ti,ab,kw OR trichoadenoma*:ti,ab,kw OR trichoblastoma*:ti,ab,kw OR trichoepithelioma*:ti,ab,kw OR tricholemmoma*:ti,ab,kw OR tumor*:ti,ab,kw OR tumour*:ti,ab,kw OR xanthoastrocytoma*:ti,ab,kw OR xanthofibroma*:ti,ab,kw OR xanthosarcoma*:ti,ab,kw \| 6823281 \| \| #14 \| 'neoplasm'/exp \| 6450685 \| \| #13 \| #1 OR #2 OR #3 OR #4 OR #5 OR #6 OR #7 OR #8 OR #9 OR #10 OR #11 OR #12 \| 2395720 \| \| #12 \| 'textbook outcome*':ti,ab,kw \| 505 \| \| #11 \| (care NEAR/2 standard*):ti,ab,kw \| 149251 \| \| #10 \| 'health care disparity'/de \| 25268 \| \| #9 \| ((countr* OR nation* OR healthcare OR health OR hospital* OR regional*) NEAR/5 (compar* OR differenc* OR disparit* OR inequit* OR inequalit* OR variation*)):ti,ab,kw \| 393935 \| \| #8 \| ((clinical OR medical) NEAR/1 audit*):ti,ab,kw \| 8493 \| \| #7 \| 'clinical audit'/de \| 58847 \| \| #6 \| (outcome NEAR/2 (assessment* OR indicator* OR measure*)):ti,ab,kw \| 420743 \| \| #5 \| 'outcome assessment'/de \| 911000 \| \| #4 \| ((quality OR performance) NEAR/2 (assessment* OR assurance OR indicator* OR measur*)):ti,ab,kw \| 215838 \| \| #3 \| 'health care quality'/de \| 286385 \| \| #2 \| benchmark*:ti,ab,kw OR 'bench mark*':ti,ab,kw OR 'best practice*':ti,ab,kw OR metric*:ti,ab,kw \| 309787 \| \| #1 \| 'benchmarking'/de \| 27468 \| | |

3. Web of Science Core Collection

| Interface: Clarivate Analytics  Editions = A&HCI , ESCI , SCI-EXPANDED , SSCI  Date of Search: 2024-05-30  Number of hits: 1536 | Field labels   - TS/Topic = title, abstract, author keywords and Keywords Plus - NEAR/x = within x words, regardless of order - * = truncation of word for alternate endings   Note: the *Exact search*-function was used for all the searches |
| --- | --- |
| \| # \| Search Query \| Results \| \| --- \| --- \| --- \| \| 1 \| TS=(Benchmark* OR bench-mark* OR "best practice*" OR metric* ) \| 699179 \| \| 2 \| TS=((quality OR performance ) NEAR/2 (assessment* OR assurance OR indicator* OR measur* )) \| 394429 \| \| 3 \| TS=(outcome NEAR/2 (assessment* OR indicator* OR measure* )) \| 332880 \| \| 4 \| TS=((clinical OR medical ) NEAR/1 audit* ) \| 6571 \| \| 5 \| TS=((countr* OR nation* OR healthcare OR health OR hospital* OR regional* ) NEAR/5 (compar* OR differenc* OR disparit* OR inequit* OR inequalit* OR variation* )) \| 414574 \| \| 6 \| TS=(care NEAR/2 standard* ) \| 81494 \| \| 7 \| TS="textbook outcome*" \| 413 \| \| 8 \| #7 OR #6 OR #5 OR #4 OR #3 OR #2 OR #1 \| 1837246 \| \| 9 \| TS=(acanthoma* OR adamantinoma* OR adenoameloblastoma* OR adenocarcinoma* OR adenofibroma* OR adenolipoma* OR adenolymphoma* OR adenoma* OR adenomyoepithelioma* OR adenomyoma* OR adenosarcoma* OR aesthesioneuroblastoma* OR ameloblastoma* OR androblastoma* OR angioblastoma* OR angiofibroma* OR angiofibrosarcoma* OR angioleiomyoma* OR angiolipoma* OR angiomyolipoma* OR angiomyoma* OR angiomyxoma* OR angiosarcoma* OR arrhenoblastoma* OR astroblastoma* OR astrocytoma* OR astroglioma* OR blastoma* OR cancer* OR carcinoid OR carcinoma* OR carcinosarcoma* OR cholangiocarcinoma* OR cholangiosarcoma* OR chondroblastoma* OR chondrosarcoma* OR chorio* OR chorioadenoma* OR choriocarcinoma* OR chorioepithelioma* OR chorionepithelioma* OR choristoma* OR comedocarcinoma* OR cystadenocarcinoma* OR cystadenofibroma* OR cystadenoma* OR cystosarcoma* OR dermatofibroma* OR dermatofibrosarcoma* OR elastofibroma* OR enchondroma* OR endothelioma* OR ependymoblastoma* OR ependymoma* OR epithelioma* OR erythroleukemia* OR esthesioneuroblastoma* OR esthesioneuroepithelioma* OR fibroadenoma* OR fibroadenosarcoma* OR fibrochondrosarcoma* OR fibroepithelioma* OR fibroid* OR fibrolipoma* OR fibroliposarcoma* OR fibromatosis OR fibromyoma* OR fibromyxolipoma* OR fibromyxoma* OR fibrosarcoma* OR fibroxanthosarcoma* OR ganglioblastoma* OR gangliocytoma* OR ganglioglioma* OR ganglioneuroblastoma* OR ganglioneurofibroma* OR ganglioneuroma* OR gastrinoma* OR germinoma* OR glioblastoma* OR gliofibroma* OR glioma* OR glioneuroma* OR gliosarcoma* OR glomangiomyoma* OR glomangiosarcoma* OR glucagonoma OR gonadoblastoma* OR gonocytoma* OR germinoblastoma* OR gynandroblastoma* OR h$emangioblastoma* OR h$emangioma* OR h$emangiopericytoma* OR h$emangiosarcoma* OR h$emartoma* OR h$emangioendotheliosarcoma* OR h$epatoblastoma* OR h$epatocarcinoma* OR h$epatocholangiocarcinoma* OR h$epatoma* OR hidradenoma* OR histiocytoma* OR hodgkin* OR hydradenoma* OR immunocytoma* OR leiomyoblastoma* OR leiomyofibroma* OR leiomyoma* OR leiomyosarcoma* OR leucocythaemia OR leucocythemia OR leukemi* OR lipoadenoma* OR lipoblastoma* OR lipoma* OR liposarcoma* OR luteoma* OR lymphangiomyoma* OR lymphangiosarcoma* OR lymphoepithelioma* OR lymphoma* OR lymphosarcoma OR malignanc* OR malignant OR masculinovoblastoma* OR mastocytoma* OR medulloblastoma* OR medullocytoma* OR medulloepithelioma* OR medullomyoblastoma* OR melanoameloblastoma* OR melanocytoma* OR melanom* OR meningioblastoma* OR mesenchymoma* OR mesothelioma* OR metastasi* OR microglioma* OR myelolipoma* OR myeloma* OR myoblastoma* OR myoepithelioma* OR myofibroblastoma* OR myofibroma* OR myofibrosarcoma* OR myolipoma* OR myoma* OR myosarcoma* OR myxofibroma* OR myxolipoma* OR myxoliposarcoma* OR myxoma* OR neoplas* OR nephroblastoma* OR neurilemmoma* OR neurilemoma* OR neuroblastoma* OR neurocytoma* OR neuroepithelioma* OR neurofibroma* OR neurofibrosarcoma* OR neurolipocytoma* OR neuroma* OR odontoameloblastoma* OR oligoastrocytoma* OR oligodendroglioma* OR oncocytoma* OR orchioblastoma* OR osteoblastoma* OR osteochondroma* OR osteochondrosarcoma* OR osteoclastoma* OR osteofibrosarcoma* OR osteosarcoma* OR pancreatoblastoma* OR paraganglioma* OR phaeochromocytoma* OR pheochromoblastoma* OR pheochromocytoma* OR pinealblastoma* OR pinealoblastoma* OR pineoblastoma* OR pineocytoma* OR plasmacytoma* OR pneumoblastoma* OR pneumocytoma* OR porocarcinoma* OR preleukemia* OR prolactinoma* OR reticulohistiocytoma* OR reticulolymphosarcoma OR retinoblastoma* OR rhabdomyoma* OR rhabdomyosarcoma* OR rhabdosarcoma* OR sarcoma* OR seminoma* OR spermatocytoma* OR spiradenoma* OR spongioblastoma* OR subependymoma* OR syringadenoma* OR syringocystadenoma* OR syringoma* OR teratocarcinoma* OR teratoma* OR thecoma* OR thymolipoma* OR thymoma* OR trichilemmoma* OR trichoadenoma* OR trichoblastoma* OR trichoepithelioma* OR tricholemmoma* OR tumor* OR tumour* OR xanthoastrocytoma* OR xanthofibroma* OR xanthosarcoma* ) \| 6125350 \| \| 10 \| TS=oncology \| 210373 \| \| 11 \| #9 OR #10 \| 6164634 \| \| 12 \| TS=((risk OR "case mix" OR case-mix OR casemix ) NEAR/3 adjust* ) \| 61816 \| \| 13 \| TS=risk-adjust* \| 16449 \| \| 14 \| #12 OR #13 \| 61976 \| \| 15 \| #8 AND #11 AND #14 \| 1536 \| | |

**Appendix 2:** Excluded studies

| **Study** | **Reason for exclusion** |
| --- | --- |
| Gomon 2023 (1) | Wrong aim. Study focuses on methods for assessing quality deviations over time, not absolute differences in hospital performance. |
| Koeter 2021 (2) | Benchmark a process indicator (sphincter preservation) |
| Visser 2023 (3) | No case-mix adjustment presented, do not present any case mix-adjustment model for an outcome or show case mix-adjusted outcome rates |
| Petrova 2019 (4) | Benign disease included |
| Brosens 2008 (5) | Review article |
| Aletti 2007 (6) | US cohort |
| Brønserud 2019 (7) | Cross-sectional study |
| Van Roessel 2020 (8) | Benign disease included |
| Koeter 2020 (9) | Process indicator benchmarked |
| Ptok 2011 (10) | Too short follow-up, in-hospital mortality |
| Raymond 2017 (11) | US cohort |
| Brunelli 2008 (12) | Too short follow-up, in-hospital mortality |
| In 2016 (13) | US cohort |
| McMillan 2016 (14) | US cohort and unclear whether the study only included pancreatic cancer surgeries or all pancreatic surgeries |
| Almoudaris 2013 (15) | No description of case-mix adjustment |
| Mosconi 2023 (16) | US cohort |
| Brunelli 2017 (17) | Not specified which cancers they are benchmarking |
| Nouraei 2013 (18) | Too short follow-up, in-hospital endpoints |
| Schouwenburg 2018 (19) | The benchmarked indicator is a process indicator (usage of adjuvant chemotherapy) |
| Elfrink 2021, June (20) | Wrong aim. They compare hospital variation in the usage of colorectal liver metastasis surgery plus ablation (a process indicator). |
| Penninckx 2013 (21) | Too short follow-up, in-hospital endpoint |
| Brunelli 2009 (22) | Too short follow-up, in-hospital mortality |
| Morris 2006 (23) | Wrong aim |
| Abel 2014 (24) | Cross-sectional design |
| Byrne 2013 (25) | Include benign diseases |
| Saunders 2014 (26) | Cross-sectional design |
| Griffiths 2002 (27) | Not specified whether they only include patients with cancer or all types of head and neck surgery. |
| Tekkis 2003 (28) | Too short follow-up, in-hospital mortality |
| Tekkis 2004 (29) | Too short follow-up, in-hospital mortality |
| Brunelli 1999 (30) | Not specified where the population is from. |
| Lagarde 2008 (31) | Aim to develop model for individual prediction |
| Ten Berge 2018 (32) | Wrong aim, aims to describe the Dutch lung surgery audit, without detailing outcomes or case-mix methods. |
| Lagarde 2007 (33) | Too short follow-up, in-hospital mortality |
| Van Dishoeck 2013 (34) | Included benign diseases |
| Van Brommel 2017 (35) | Aim to describe the Dutch lung surgery audit, without detailing outcomes or case-mix methods. |
| Tsilimigras 2020 (36) | Cohorts outside Europe |
| Aquina 2021 (37) | US cohort |
| De Graaff 2024 (38) | Wrong aim, do not present hospital/regional outcome variations but general trends over time. |
| Van der Heiden 2015 (39) | Wrong aim, aiming to evaluate a quality metric for benchmarking |
| Harrison 2013 (40) | Wrong aim, aiming to assess how socioeconomic status impact colorectal cancer survival, applying multilevel latent class modelling. |
| Masum 2022 (41) | Wrong aim, developing models for individual outcome prediction using ML techniques. |
| Laios 2022 (42) | Wrong aim, model development for individual prediction of hospital LOS |
| De Graaff 2024 (43) | Benchmark a process indication, repeated liver resection |
| Olthof 2019 (44) | Cohort from Asia |
| Garcia-Torecillas 2020 (45) | Too short follow-up, in-hospital mortality |
| Die Loucou 2020 (46) | Wrong aim, model development for individual risk prediction |
| Warwick 2013 (47) | Wrong aim, the aim of the study is to explain observed differences in postoperative survival rather than identify the differences. |
| Cowling 2020 (48) | Wrong aim, model development for individual prediction |
| Sherlaw-Johnson 2008 (49) | Cross-sectional study design |
| Wouters 2010 (50) | Benchmarked process indicators |
| Nur 2015 (51) | Wrong aim. Assessing the relationship between low rates of surgical resection of lung cancer and 1-year mortality rates between units. |
| Olthof 2024 (52) | Wrong aim. Looking at factors predicting failure to rescue |

**Full references of excluded studies**

1. Gomon D, Sijmons J, Putter H, Dekker JW, Tollenaar R, Wouters M, et al. Inspecting the quality of care: a comparison of CUSUM methods for inter hospital performance. HEALTH SERVICES AND OUTCOMES RESEARCH METHODOLOGY. 2023.

2. Koeter T, de Nes LCF, Wasowicz DK, Zimmerman DDE, Verhoeven RHA, Elferink MA, et al. Hospital variation in sphincter-preservation rates in rectal cancer treatment: results of a population-based study in the Netherlands. BJS open. 2021;5(4).

3. Visser MR, Straatman J, Voeten DM, Gisbertz SS, Ruurda JP, Luyer MDP, et al. Hospital Variation in Feeding Jejunostomy Policy for Minimally Invasive Esophagectomy: A Nationwide Cohort Study. Nutrients. 2023;15(1).

4. Petrova E, Lapshyn H, Bausch D, D'Haese J, Werner J, Klier T, et al. Risk stratification for postoperative pancreatic fistula using the pancreatic surgery registry StuDoQ|Pancreas of the German Society for General and Visceral Surgery. Pancreatology : official journal of the International Association of Pancreatology (IAP) [et al]. 2019;19(1):17-25.

5. Brosens RPM, Oomen JLT, Cuesta MA, Engel AF. Scoring Systems for Prediction of Outcome in Colon and Rectal Surgery. Seminars in Colon and Rectal Surgery. 2008;19(1):53-61.

6. Aletti GD, Santillan A, Eisenhauer EL, Hu J, Aletti G, Podratz KC, et al. A new frontier for quality of care in gynecologic oncology surgery: multi-institutional assessment of short-term outcomes for ovarian cancer using a risk-adjusted model. Gynecol Oncol. 2007;107(1):99-106.

7. Brønserud MM, Iachina M, Green A, Groenvold M, Jakobsen E. Patient reported outcome data as performance indicators in surgically treated lung cancer patients. Lung Cancer. 2019;130:143-8.

8. van Roessel S, Mackay TM, van Dieren S, van der Schelling GP, Nieuwenhuijs VB, Bosscha K, et al. Textbook Outcome: Nationwide Analysis of a Novel Quality Measure in Pancreatic Surgery. Annals of surgery. 2020;271(1):155-62.

9. Koeter T, Elferink MA, Verhoeven RHA, Zimmerman DDE, Wasowicz DK, Verheij M, et al. Hospital variance in neoadjuvant rectal cancer treatment and the influence of a national guideline update: Results of a nationwide population-based study. Radiotherapy and oncology : journal of the European Society for Therapeutic Radiology and Oncology. 2020;145:162-71.

10. Ptok H, Marusch F, Schmidt U, Gastinger I, Wenisch HJC, Lippert H. Risk adjustment as basis for rational benchmarking: the example of colon carcinoma. World journal of surgery. 2011;35(1):196-205.

11. Raymond DP. Risk Adjustment and Performance Measurement for Lung Cancer Resection. Thorac Surg Clin. 2017;27(3):215-20.

12. Brunelli A, Varela G, Van Schil P, Salati M, Novoa N, Hendriks JM, et al. Multicentric analysis of performance after major lung resections by using the European Society Objective Score (ESOS). Eur J Cardiothorac Surg. 2008;33(2):284-8.

13. In H, Palis BE, Merkow RP, Posner MC, Ferguson MK, Winchester DP, et al. Doubling of 30-Day Mortality by 90 Days After Esophagectomy: A Critical Measure of Outcomes for Quality Improvement. Annals of surgery. 2016;263(2):286-91.

14. McMillan MT, Soi S, Asbun HJ, Ball CG, Bassi C, Beane JD, et al. Risk-adjusted Outcomes of Clinically Relevant Pancreatic Fistula Following Pancreatoduodenectomy: A Model for Performance Evaluation. Annals of surgery. 2016;264(2):344-52.

15. Almoudaris AM, Burns EM, Bottle A, Aylin P, Darzi A, Vincent C, et al. Single measures of performance do not reflect overall institutional quality in colorectal cancer surgery. Gut. 2013;62(3):423-9.

16. Mosconi C, O'Rourke J, Kloeckner R, Sturm L, Golfieri R, Celsa C, et al. Textbook Outcome After Trans-arterial Chemoembolization for Hepatocellular Carcinoma. Cardiovascular and interventional radiology. 2023;46(4):449-59.

17. Brunelli A, Salati M, Rocco G, Varela G, Van Raemdonck D, Decaluwe H, et al. European risk models for morbidity (EuroLung1) and mortality (EuroLung2) to predict outcome following anatomic lung resections: an analysis from the European Society of Thoracic Surgeons database. European journal of cardio-thoracic surgery : official journal of the European Association for Cardio-thoracic Surgery. 2017;51(3):490-7.

18. Nouraei SAR, Middleton SE, Hudovsky A, Darzi A, Stewart S, Kaddour H, et al. A national analysis of the outcome of major head and neck cancer surgery: implications for surgeon-level data publication. Clinical otolaryngology : official journal of ENT-UK ; official journal of Netherlands Society for Oto-Rhino-Laryngology & Cervico-Facial Surgery. 2013;38(6):502-11.

19. Schouwenburg MG, Busweiler LAD, Beck N, Henneman D, Amodio S, van Berge Henegouwen MI, et al. Hospital variation and the impact of postoperative complications on the use of perioperative chemo(radio)therapy in resectable gastric cancer. Results from the Dutch Upper GI Cancer Audit. European journal of surgical oncology : the journal of the European Society of Surgical Oncology and the British Association of Surgical Oncology. 2018;44(4):532-8.

20. Elfrink AKE, Nieuwenhuizen S, van den Tol MP, Burgmans MC, Prevoo W, Coolsen MME, et al. Hospital variation in combined liver resection and thermal ablation for colorectal liver metastases and impact on short-term postoperative outcomes: a nationwide population-based study. HPB. 2021;23(6):827-39.

21. Penninckx F, Fieuws S, Beirens K, Demetter P, Ceelen W, Kartheuser A, et al. Risk adjusted benchmarking of abdominoperineal excision for rectal adenocarcinoma in the context of the Belgian PROCARE improvement project. Gut. 2013;62(7):1005-11.

22. Brunelli A, Berrisford RG, Rocco G, Varela G. The European Thoracic Database project: composite performance score to measure quality of care after major lung resection. European journal of cardio-thoracic surgery : official journal of the European Association for Cardio-thoracic Surgery. 2009;35(5):769-74.

23. Morris E, Haward RA, Gilthorpe MS, Craigs C, Forman D. The impact of the Calman-Hine report on the processes and outcomes of care for Yorkshire's colorectal cancer patients. British journal of cancer. 2006;95(8):979-85.

24. Abel GA, Saunders CL, Lyratzopoulos G. Cancer patient experience, hospital performance and case mix: evidence from England. Future Oncol. 2014;10(9):1589-98.

25. Byrne BE, Mamidanna R, Vincent CA, Faiz O. Population-based cohort study comparing 30- and 90-day institutional mortality rates after colorectal surgery. British Journal of Surgery. 2013;100(13):1810-7.

26. Saunders CL, Abel GA, Lyratzopoulos G. What explains worse patient experience in London? Evidence from secondary analysis of the Cancer Patient Experience Survey. BMJ open. 2014;4(1):e004039.

27. Griffiths H, Cuddihy P, Davis S, Parikh S, Tomkinson A. Risk-adjusted comparative audit. Is Possum applicable to head and neck surgery? Clin Otolaryngol Allied Sci. 2002;27(6):517-20.

28. Tekkis PP, McCulloch P, Steger AC, Benjamin IS, Poloniecki JD. Mortality control charts for comparing performance of surgical units: validation study using hospital mortality data. Bmj. 2003;326(7393):786-8.

29. Tekkis PP, Prytherch DR, Kocher HM, Senapati A, Poloniecki JD, Stamatakis JD, et al. Development of a dedicated risk-adjustment scoring system for colorectal surgery (colorectal POSSUM). Br J Surg. 2004;91(9):1174-82.

30. Brunelli A, Fianchini A, Gesuita R, Carle F. POSSUM scoring system as an instrument of audit in lung resection surgery. Physiological and operative severity score for the enumeration of mortality and morbidity. Ann Thorac Surg. 1999;67(2):329-31.

31. Lagarde SM, Reitsma JB, Maris AK, van Berge Henegouwen MI, Busch OR, Obertop H, et al. Preoperative prediction of the occurrence and severity of complications after esophagectomy for cancer with use of a nomogram. Ann Thorac Surg. 2008;85(6):1938-45.

32. Ten Berge M, Beck N, Heineman DJ, Damhuis R, Steup WH, van Huijstee PJ, et al. Dutch Lung Surgery Audit: A National Audit Comprising Lung and Thoracic Surgery Patients. Ann Thorac Surg. 2018;106(2):390-7.

33. Lagarde SM, Maris AK, de Castro SM, Busch OR, Obertop H, van Lanschot JJ. Evaluation of O-POSSUM in predicting in-hospital mortality after resection for oesophageal cancer. Br J Surg. 2007;94(12):1521-6.

34. van Dishoeck AM, Koek MB, Steyerberg EW, van Benthem BH, Vos MC, Lingsma HF. Use of surgical-site infection rates to rank hospital performance across several types of surgery. Br J Surg. 2013;100(5):628-36; discussion 37.

35. van Bommel AC, Spronk PE, Vrancken Peeters MT, Jager A, Lobbes M, Maduro JH, et al. Clinical auditing as an instrument for quality improvement in breast cancer care in the Netherlands: The national NABON Breast Cancer Audit. J Surg Oncol. 2017;115(3):243-9.

36. Tsilimigras DI, Mehta R, Merath K, Bagante F, Paredes AZ, Farooq A, et al. Hospital variation in Textbook Outcomes following curative-intent resection of hepatocellular carcinoma: an international multi-institutional analysis. HPB (Oxford). 2020;22(9):1305-13.

37. Aquina CT, Hamad A, Becerra AZ, Cloyd JM, Tsung A, Pawlik TM, et al. Is Textbook Oncologic Outcome a Valid Hospital-Quality Metric after High-Risk Surgical Oncology Procedures? Ann Surg Oncol. 2021;28(13):8028-45.

38. de Graaff MR, Klaase JM, Dulk MD, Buis CI, Derksen WJM, Hagendoorn J, et al. Outcomes of liver surgery: A decade of mandatory nationwide auditing in the Netherlands. Eur J Surg Oncol. 2024;50(6):108264.

39. van der Heiden-van der Loo M, Siesling S, Wouters MW, van Dalen T, Rutgers EJ, Peeters PH. The Value of Ipsilateral Breast Tumor Recurrence as a Quality Indicator: Hospital Variation in the Netherlands. Ann Surg Oncol. 2015;22 Suppl 3:S522-8.

40. Harrison WJ, Gilthorpe MS, Downing A, Baxter PD. Multilevel Latent Class Modelling of Colorectal Cancer Survival Status at Three Years and Socioeconomic Background Whilst Incorporating Stage of Disease. International Journal of Statistics and Probability. 2013;2(3):85-undefined.

41. Masum S, Hopgood A, Stefan S, Flashman K, Khan J. Data analytics and artificial intelligence in predicting length of stay, readmission, and mortality: a population-based study of surgical management of colorectal cancer. Discov Oncol. 2022;13(1):11.

42. Laios A, De Freitas DLD, Saalmink G, Tan YS, Johnson R, Zubayraeva A, et al. Stratification of Length of Stay Prediction following Surgical Cytoreduction in Advanced High-Grade Serous Ovarian Cancer Patients Using Artificial Intelligence; the Leeds L-AI-OS Score. Curr Oncol. 2022;29(12):9088-104.

43. de Graaff MR, Klaase JM, den Dulk M, Te Riele WW, Hagendoorn J, van Heek NT, et al. Hospital variation and outcomes after repeat hepatic resection for colorectal liver metastases: a nationwide cohort study. HPB (Oxford). 2024;26(6):789-99.

44. Olthof PB, Miyasaka M, Koerkamp BG, Wiggers JK, Jarnagin WR, Noji T, et al. A comparison of treatment and outcomes of perihilar cholangiocarcinoma between Eastern and Western centers. HPB (Oxford). 2019;21(3):345-51.

45. Garcia-Torrecillas JM, Olvera-Porcel MC, Ferrer-Marquez M, Rosa-Garrido C, Rodriguez-Barranco M, Lea-Pereira MC, et al. Predictive Model of the Risk of In-Hospital Mortality in Colorectal Cancer Surgery, Based on the Minimum Basic Data Set. Int J Environ Res Public Health. 2020;17(12):4216-undefined.

46. Die Loucou J, Pages PB, Falcoz PE, Thomas PA, Rivera C, Brouchet L, et al. Validation and update of the thoracic surgery scoring system (Thoracoscore) risk model. Eur J Cardiothorac Surg. 2020;58(2):350-6.

47. Warwick J, Will O, Allgood P, Miller R, Duffy S, Greenberg D. Variation in colorectal cancer treatment and survival: a cohort study covering the East Anglia region. Colorectal Dis. 2013;15(10):1243-52.

48. Cowling TE, Bellot A, Boyle J, Walker K, Kuryba A, Galbraith S, et al. One-year mortality of colorectal cancer patients: development and validation of a prediction model using linked national electronic data. Br J Cancer. 2020;123(10):1474-80.

49. Sherlaw-Johnson C, Datta P, McCarthy M. Hospital differences in patient satisfaction with care for breast, colorectal, lung and prostate cancers. Eur J Cancer. 2008;44(11):1559-65.

50. Wouters MW, Siesling S, Jansen-Landheer ML, Elferink MA, Belderbos J, Coebergh JW, et al. Variation in treatment and outcome in patients with non-small cell lung cancer by region, hospital type and volume in the Netherlands. Eur J Surg Oncol. 2010;36 Suppl 1:S83-92.

51. Nur U, Quaresma M, De Stavola B, Peake M, Rachet B. Inequalities in non-small cell lung cancer treatment and mortality. J Epidemiol Community Health. 2015;69(10):985-92.

52. Olthof PB, Bouwense SAW, Bednarsch J, Dewulf M, Kazemier G, Maithel S, et al. Failure to Rescue After Resection of Perhilar Cholangiocarcinoma in an International Multicenter Cohort. Ann Surg Oncol. 2024.

**Appendix 3:** Quality assessment of included studies

|  | **Selection** | | | | **Comparability** | **Outcome** | | | **Fit inclusion/ exclusion critiera?** | **Decision** |
| --- | --- | --- | --- | --- | --- | --- | --- | --- | --- | --- |
| Studies | Representativeness of the exposed cohort | Selection of the non-exposed cohort | Ascertainment of exposure | Showing that outcome was not present at study start | Comparability (Y=YES, S=Some extent, N=No) | Assessment of outcome | Adequacy of follow-up of cohorts | Missing data | Assessment |  |
| De Graaff 2023 | x | x | x | x | **Description: S**, describe the main analyses (propensity score matching well, but not how they built the case-mix adjustment model) **Appropriate: Y Assumptions: Y** **Confounder identification: Y Confounder measure: Y Sensitivity: N Model characteristics: S**, only for the propensity score matching  **Believe results: Y** | x | x | >25 % missing data in two case-mix variables. They were excluded. | YES | INCLUDE |
| Kowalski 2022 | (x), they included patients from certified hospitals which perform half of the colorectal surgeries in Germany | x | x | x | **Description: Y Appropriate: Y Assumptions: Y Confounder identification: Y Confounder measure: Y** **Sensitivity: Y  Model characteristics: S**, they describe that they calculate R^2^ and do a 10-fold cross-validation but do not show the results. **Believe results: Y** | x | x | 75 % response rate on 12 months follow-up. Missing data is managed by multiple imputation. | YES | INCLUDE |
| Nilssen 2024 | x | x | x | x | **Description: S**, quite shallow regarding the case-mix adjustment modelling. **Appropriate: Y Assumptions: Y Confounder identification: S**, comorbidity burden would be appropriate to test in the model. **Confounder measure: Y Sensitivity: N**  **Model characteristics: N Believe results: Y** | x | x | x | YES | INCLUDE |
| Thurell 2023 | (x), the regions consisted of one third of the total population. | x | x | x | **Description: Y**  **Appropriate: Y Assumptions: Y Confounder identification: S**, comorbidity burden and performance status would have been desirable.  **Confounder measure: Y Sensitivity: N**  **Model characteristics: N Believe results: Y** | x | x | Report the amount of missing data in each variable but not how missing data were handled. | YES | INCLUDE |
| Govaert 2016 | (x), recruited hospitals represented one third of the total population | x | x | x | **Description: Y  Appropriate: S,** they included all significant variables from the univariable analyses in the multivariable analyses. Risk of overfitting. **Assumptions: Y Confounder identification: Y Confounder measure: Y** **Sensitivity: N  Model characteristics: N Believe results: Y** | x | x | Analysed variables with more than 5 % missing data separately, | YES | INCLUDE |
| Gillthorpe 2011 | x | x | x | x | **Description: Y  Appropriate: Y Assumptions: Y Confounder identification: Y Confounder measure: Y Sensitivity: Y**, multilevel modelling and comparing ranking with another statistical approach.  **Model characteristics: N Believe results: Y** | x | x | Stage had 13.4 % missing data. They managed this by incorporating a stage missing category in the model. | YES | INCLUDE |
| Algera 2023, july | x | x | x | x | **Description: S**, could describe in further detail the regression strategy.  **Appropriate: Y Assumptions: Y Confounder identification: Y Confounder measure: Y Sensitivity: N**  **Model characteristics: N Believe results: Y** | x | x | x, multiple imputation | YES | INCLUDE |
| Ten Berge 2021 | x | x | x | x | **Description: S**, they do not specify the tested confounders. They furthermore did not specify the “multicollinearity test“ used.  **Appropriate: Y Assumptions: Y Confounder identification: S Confounder measure: Y Sensitivity: N Model characteristics: N Believe results: S**, they are not presenting the final case-mix adjustment model, meaning that it is hard to assess the validity of the results. | x | x | x  Excluded missing data if variable had<5% missing data. Otherwise reported as a separate analyses. | YES | INCLUDE |
| Tighe 2023 | Only using data from three hospitals which might not be representative for all of UK. | x | x | x | **Description: Y  Appropriate: Y Assumptions: Y Confounder identification: S**, lacking certain confounders such as age, comorbidity burden and performance status.  **Confounder measure: Y** **Sensitivity: Y**, cross-validation **Model characteristics: Y Believe results: Y** | x | x | Displaying missing data but no description of how it was managed. | YES | INCLUDE |
| Fischer 2016 | x | x | x | x | **Description: Y**  **Appropriate: Y Assumptions: Y Confounder identification: Y  Confounder measure: Y Sensitivity: Y**, bootstrapping  **Model characteristics: Y Believe results: Y** | x | x | Multiple imputation | YES | INCLUDE |
| Damhuis (2006) | x | x | x | x | **Description: S**, could go into further depth of regression model development. **Appropriate: Y Assumptions: Y Confounder identification: S**, would be valuable to include TNM-stage. Unclear why they included type of surgery.  **Confounder measure: Y Sensitivity: Y**, described how chance impact the results  **Model characteristics: Y Believe results: Y** | x | x | Not described | YES | INCLUDE |
| Kolfschoten 2013 | x | x | x | x | **Description: Y  Appropriate: Y Assumptions: Y Confounder identification: Y  Confounder measure: Y Sensitivity: N Model characteristics: Y Believe results: Y** | x | x | x  Missing data was analysed in a separate category | YES | INCLUDE |
| Boyle 2023 | x | x | x | x | **Description: Y  Appropriate: Y Assumptions: Y Confounder identification: Y  Confounder measure: Y Sensitivity: Y, calibration. Power calculation. Model characteristics: Y Believe results: S**, the risk-adjustment model has a low C-statistic (0.58). | x | x | Not described | YES | INCLUDE |
| Tighe 2021 | x | x | x | x | **Description: S**, quite shallow explanation of the tested methods and included independent variables. **Appropriate: S**, due to above-mentioned lack of description **Assumptions: Y Confounder identification: S**, case-complexity defined as surgical duration is debatable to use as a case-mix factor.  **Confounder measure: Y Sensitivity: Y**, cross-validation in 10-fold for internal validation. **Model characteristics: Y Believe results: S**, they changed the outcome definition to get a better model fit which might be problematic. | x | x | Not described | YES | INCLUDE |
| Talsma 2014 | x | x | x | x | **Description: N**, very shallow description of the case-mix model development.  **Appropriate: Y Assumptions: Y Confounder identification: N**, they use usage of neoadjuvant therapy, surgery type and resection margins as case-mix factors in the model. **Confounder measure: Y Sensitivity: N Model characteristics: N Believe results: N**, not possible to evaluate the model as they neither display interhospital variations nor model performance metrics. | x | x | x, multiple imputation | YES | INCLUDE |
| Walker 2015 | x | x | x | x | **Description: Y  Appropriate: Y Assumptions: Y Confounder identification: Y  Confounder measure: Y Sensitivity: Y Model characteristics: Y Believe results: Y** | x | x | x, multiple imputation. | YES | INCLUDE |
| Tighe 2017, June | x | x | x | x | **Description: N Appropriate: Y Assumptions: Y Confounder identification: Y Confounder measure: Y Sensitivity: Y Model characteristics: Y Believe results: Y** | x | x | Showed missing data in each variable but not described how missing data was managed. | YES | INCLUDE |
| McArdle 2002 | x | x | x | x | **Description: N Appropriate: Y Assumptions: Y Confounder identification: Y Confounder measure: N Sensitivity: N Model characteristics: N Believe results: Y** | x | x | No description | YES | INCLUDE |
| Matthes-Martin 2008 | x | x | x | x | **Description: Y Appropriate: Y Assumptions: Y Confounder identification: Y Confounder measure: Y Sensitivity: Y Model characteristics: Y Believe results: Y** | x | x | x | YES | INCLUDE |
| Talsma 2011 | x | x | x | x | **Description: Y Appropriate: Y Assumptions: Y Confounder identification: S**, no comorbidity burden or performance status. **Confounder measure: Y Sensitivity: S**, presented ROC-curve which gives a sense of overall model sensitivity. They did not develop several models and see how the performance metrics changed. **Model characteristics: Y Believe results: Y** | x | x | No description | YES | INCLUDE |
| Beck 2018 | x | x | x | x | **Description: S Appropriate: Y Assumptions: Y Confounder identification: Y Confounder measure: Y Sensitivity: N Model characteristics: N Believe results: Y** | x | x | x | YES | INCLUDE |
| Elfrink 2022 | x | x | x | x | **Description: Y** **Appropriate: Y Assumptions: Y Confounder identification: Y Confounder measure: Y Sensitivity: N Model characteristics: N Believe results: Y** | x | x | x, reported separately | YES | INCLUDE |
| Bernard 2011 | x | x | x | x | **Description: Y Appropriate: Y Assumptions: Y Confounder identification: Y Confounder measure: Y Sensitivity: S Model characteristics: Y Believe results: Y** | x | x | x multiple imputation | YES | INCLUDE |
| Nickelsen 2005 | x | x | x | x | **Description: S Appropriate: Y Assumptions: S Confounder identification: N Confounder measure: N Sensitivity: N Model characteristics: N Believe results: Y** | x | x | x | YES | INCLUDE |
| Algera 2023, April | x | x | x | x | **Description: Y Appropriate: N  Assumptions: Y Confounder identification: S**, include both a process indicator and an outcome indicator in the case-mix adjustment model. **Confounder measure: Y Sensitivity: N Model characteristics: N Believe results: N** | x | x | x  Excluded data if less than 5 % was missing. Otherwise presented as a separate category, | YES | INCLUDE |
| Salet 2023 | x | x | x | x | **Description: Y Appropriate: Y Assumptions: Y Confounder identification: Y Confounder measure: Y Sensitivity: Y Model characteristics: Y Believe results: Y** | x | x | No description | YES | INCLUDE |
| Van der Heiden 2012 | x | x | x | x | **Description: Y Appropriate: Y Assumptions: S Confounder identification: S Confounder measure: N Sensitivity: N Model characteristics: N Believe results: Y** | x | x | No description | YES | INCLUDE |
| Busweiler 2017 | x | x | x | x | **Description: Y Appropriate: Y Assumptions: Y Confounder identification: S Confounder measure: N Sensitivity: N Model characteristics: N Believe results: Y** | x | x | x | YES | INCLUDE |
| Voeten 2020 | X | x | x | x | **Description: Y Appropriate: Y Assumptions: Y Confounder identification: N Confounder measure: N Sensitivity: N Model characteristics: N Believe results: Y** | x | x | x | YES | INCLUDE |
| Snijders 2013 | x | x | x | x | **Description: Y Appropriate: Y Assumptions: Y Confounder identification: S Confounder measure: N Sensitivity: N Model characteristics: N Believe results: Y** | x | x | x | YES | INCLUDE |
| Tighe 2019, Oct | No, only four hospitals | x | x | x | **Description: N Appropriate: Y Assumptions: Y Confounder identification: N Confounder measure: N Sensitivity: Y Model characteristics: Y Believe results: Y** | x | x | Not described | YES | INCLUDE |
| Morris 2011 | x | x | x | x | **Description: Y Appropriate: Y Assumptions: Y Confounder identification: Y Confounder measure: Y Sensitivity: N Model characteristics: N Believe results: Y** | x | x | x | YES | INCLUDE |
| Elfrink 2021 oct | x | x | x | x | **Description: Y Appropriate: S**, they aim to develop and validate a risk-adjustment model. This requires model performance metrics such as discrimination and calibration. **Assumptions: Y Confounder identification: S**, inclusion of neoadjuvant chemotherapy is debatable.  **Confounder measure: Y Sensitivity: Y**, added hospital volume to the model to see whether the model changed.  **Model characteristics: N Believe results: S**, they have not shown how their model performed. | x | x | x | YES | INCLUDE |
| Henneman 2014 | x | x | x | x | **Description: Y Appropriate: Y Assumptions: Y Confounder identification: Y Confounder measure: Y Sensitivity: Y Model characteristics: N Believe results: Y** | x | x | No description | YES | INCLUDE |
| Tighe 2019, nov, LOS | No, they have patients from five hospitals in the UK | x | x | x | **Description: Y Appropriate: Y Assumptions: Y Confounder identification: S Confounder measure: Y Sensitivity: N Model characteristics: Y Believe results: Y** | x | x | Excluded cases that missed “pertinent data” | YES | INCLUDE |
| Tighe 2019, nov validation | No, only four hospitals | x | x | x | **Description: Y Appropriate: Y Assumptions: Y Confounder identification: S Confounder measure: Y Sensitivity: Y Model characteristics: Y Believe results: Y** | x | x | No description | YES | INCLUDE |
| Tighe 2014, dec | x | Not the same calendar years. | They have collected data retrospectively from site A and both retro- and prospectively from site B and C. | x | **Description: S  Appropriate: Y Assumptions: Y Confounder identification: N Confounder measure: N Sensitivity: Y Model characteristics: Y Believe results: Y** | x | x | Describe missing data in variables but not how missing data has been statistically managed | YES | INCLUDE |
| Kolfschoten 2011 | x | x | x | x | **Description: Y Appropriate: S**, they have not controlled the model for overfitting. **Assumptions: Y Confounder identification: S**, they use several process indicators as confounders. **Confounder measure: Y Sensitivity: N Model characteristics: Y Believe results: Y** | x | x | Not described | YES | INCLUDE |
| Jack 2003 | x | x | x | x | **Description: Y** **Appropriate: N**, included very few confounders. And none of them were significant predictors in the model of survival. Also included active treatment without specifying the treatment.  **Assumptions: Y Confounder identification: N Confounder measure: S Sensitivity: N Model characteristics: Y Believe results: N** | x | x | 21 % missing cases as they excluded patients which they only had death certificate information on. | YES | INCLUDE |
| Fischer 2015 | x | x | x | x | **Description: Y  Appropriate: Y Assumptions: Y Confounder identification: Y Confounder measure: Y Sensitivity: Y Model characteristics: Y Believe results: Y** | x | x | x | YES | INCLUDE |
| Tighe 2014, sept | x | No, only data from three hospitals | x | x | **Description: Y  Appropriate: Y Assumptions: Y Confounder identification: S**, including operative blood loss in the model is debatable. **Confounder measure: Y Sensitivity: N Model characteristics: Y Believe results: Y** | x | x | Not described | YES | INCLUDE |
| Das 2006 | x | No, only one centre | x | x | **Description: Y  Appropriate: S**, few patients. **Assumptions: Y Confounder identification: Y Confounder measure: Y Sensitivity: Y Model characteristics: Y Believe results: Y** | x | x | Not described | YES | INCLUDE |
| Vos 2020 (1/2) | x | x | x | x | **Description: Y  Appropriate: Y Assumptions: Y Confounder identification: Y Confounder measure: Y Sensitivity: Y Model characteristics: Y Believe results: Y** | x | x | x | YES | INCLUDE |
| Vos 2020 (2/2) | x | x | x | x | **Description: Y  Appropriate: Y Assumptions: Y Confounder identification: Y Confounder measure: Y Sensitivity: Y Model characteristics: Y Believe results: Y** | x | x | x | YES | INCLUDE |
| Tighe 2022 | x | x | x | x | **Description: Y  Appropriate: Y Assumptions: Y Confounder identification: S Confounder measure: S Sensitivity: Y Model characteristics: Y Believe results: Y** | x | x | Not described | YES | INCLUDE |
| Tighe 2017 | x | x | x | x | **Description: Y  Appropriate: Y Assumptions: Y Confounder identification: S Confounder measure: S Sensitivity: Y Model characteristics: Y Believe results: Y** | x | x | x | YES | INCLUDE |
| Sibert 2021 | x | x | x | x | **Description: Y  Appropriate: Y Assumptions: Y Confounder identification: Y Confounder measure: Y Sensitivity: S Model characteristics: Y**, R2-values ranging from 0.11 to 0.22. Low discrimination.  **Believe results: S**, the method is robust but the low discriminatory ability could mean either that the PRO domains are to a low extent influenced by case-mix or that there are other confounders not included. | x | x | x | YES | INCLUDE |
| Wahba 2023 | x | x | x | x | **Description: Y  Appropriate: Y Assumptions: Y Confounder identification: Y Confounder measure: Y Sensitivity: Y Model characteristics: Y Believe results: Y** | x | x | x | YES | INCLUDE |
| Elfrink 2021, april | x | x | x | x | **Description: Y  Appropriate: Y Assumptions: Y Confounder identification: S**, they included process indicators such as previous chemotherapy in the model. **Confounder measure: Y Sensitivity: Y**, checked multicollinearity **Model characteristics: N Believe results: Y** | x | x | Included variables with more than 5 % missing data. | YES | INCLUDE |
| Gildea 2016 | x | x | x | x | **Description: Y  Appropriate: Y Assumptions: Y Confounder identification: S**, unclear for example why stage IV patients are not excluded **Confounder measure: Y Sensitivity: N Model characteristics: S**, they show model calibration but not discrimination **Believe results: N** | x | x | Only 52 % of the patients had complete data on all variables. 42 % missing data in tumor stage and 21 % in tumor grade. High risk of bias even though they use robust strategies for managing missing data. | YES | INCLUDE |
| Henneman (2013) | x | x | x | x | **Description: Y  Appropriate: Y Assumptions: Y Confounder identification: S**, describe the variables but do not specify how they chose candidate independent variables for each model. Do not explain their choice to include neoadjuvant treatment as a case-mix factor. **Confounder measure: Y Sensitivity: N Model characteristics: N Believe results: Y** | x | x | Not described, however the registry has according to the authors almost full coverage in all variables. | YES | INCLUDE |
| Burnell (2016) | x | x | x | x | **Description: Y  Appropriate: Y Assumptions: Y Confounder identification: Y Confounder measure: Y Sensitivity: Y Model characteristics: Y Believe results: Y** | x | x | They excluded cases with missing data but did not explain why. | YES | INCLUDE |
| De Graaff (2022) | x | x | x | x | **Description: Y  Appropriate: Y Assumptions: Y Confounder identification: Y Confounder measure: Y Sensitivity: Y Model characteristics: Y Believe results: Y** | x | x | x | YES | INCLUDE |
| Voeten 2021 | No, excluding patients with postoperative complications makes it harder for the hospital to affect the outcome by improving the quality of care. | x | x | x | **Description: Y  Appropriate: N**, dividing the continuous outcome length of stay into two groups separated by the median means that the outcome was defined after initial data analyses. **Assumptions: Y Confounder identification: S**, they use several process indicators as case-mix variables. **Confounder measure: Y Sensitivity: Y**, multicollinearity and also including patients with complications. **Model characteristics: N Believe results: N**, the case mix adjustment model has limitations. | x | x | x  Missing data was analysed as a separate category if it exceeded 5 %. | YES | INCLUDE |
| Gray (2021) | x | x | x | x | **Description: S**, they could have described the regression modelling a little more thoroughly.  **Appropriate: S Assumptions: S Confounder identification: Y Confounder measure: Y Sensitivity: N Model characteristics: N Believe results: Y** | x | x | x | YES | INCLUDE |
| Greidanus (2024) | x | x | x | x | **Description: Y  Appropriate: Y**, but could have presented 3SD outliers in addition to 2SD in the funnel plot as they are comparing many hospitals. **Assumptions: Y Confounder identification: Y Confounder measure: Y Sensitivity: S** **Model characteristics: N**, no presentation of discrimination or calibration.  **Believe results: Y** | x | x | x | YES | INCLUDE |
| Aravani 2016 | x | x | x | x | **Description: Y  Appropriate: Y  Assumptions: Y Confounder identification: Y Confounder measure: Y Sensitivity: Y Model characteristics: N Believe results: Y** | x | x | x, multiple imputation model | YES | INCLUDE |
| D’Journo 2017 | x | x | x | x | **Description: Y  Appropriate: Y  Assumptions: Y Confounder identification: Y Confounder measure: Y Sensitivity: Y**, describe three different outcomes. **Model characteristics: Y Believe results: Y** | x | x | Not described, neither the amount of missing data nor how it was handled. | YES | INCLUDE |
| Van den Bosch | x | x | x | x | **Description: Y  Appropriate: Y  Assumptions: Y Confounder identification: Y Confounder measure: Y Sensitivity: N Model characteristics: S**, describe discrimination but not calibration. **Believe results: Y** | x | x | x | YES | INCLUDE |
| Damhuis 2015 | x | x | x | x | **Description: S**, no clear description of their choice of case-mix factors. **Appropriate: Y**  **Assumptions: Y Confounder identification: N**, they lack potentially important confounders such as comorbidity burden and performance status. They include type of surgery as a case-mix factor. **Confounder measure: Y Sensitivity: N Model characteristics: N Believe results: S** | x | x | x, complete case analyses. | YES | INCLUDE |
| Warps 2021 | x | x | x | x | **Description: S**, not described how they developed the model.  **Appropriate: Y  Assumptions: Y Confounder identification: S**, included surgical procedure and type of hospital In the model. **Confounder measure: Y Sensitivity: N Model characteristics: N Believe results: Y** | x | x | Not described. | YES | INCLUDE |
| Bernard 2023 | x | x | x | x | **Description: Y Appropriate: Y  Assumptions: Y Confounder identification: S**, included surgical procedure and extent of operation in the model. **Confounder measure: Y Sensitivity: N Model characteristics: N Believe results: Y** | x | x | Not described. | YES | INCLUDE |
| Blake 2024 | x | x | x | x | **Description: Y Appropriate: Y  Assumptions: Y Confounder identification: Y Confounder measure: Y Sensitivity: Y Model characteristics: Y Believe results: Y** |  |  | Describing missing data and using multiple imputation. | YES | INCLUDE |
| Myrdal 2009 | x | x | x | x | **Description: Y Appropriate: Y  Assumptions: Y Confounder identification: Y Confounder measure: Y Sensitivity: Y Model characteristics: Y Believe results: Y** | x | x | Complete case analyses. They do not present the distribution of missing data. | YES | INCLUDE |
| Skyrud 2015 | x | x | x | x | **Description: Y Appropriate: Y  Assumptions: Y Confounder identification: Y Confounder measure: Y Sensitivity: N Model characteristics: Y Believe results: Y** | x | x | Multiple imputation for tumor stage and household income. No comparison of non-imputed versus imputed models. | YES | INCLUDE |

**Appendix 4:** Description of included studies

| **Author** | **Year** | **Country** | **Cancer** | **Aim** | **Study design** | **Modelling method** | **Number of cases** | | **Outcome** | | **Case-mix factors** | | **Validation** | **Performance metrics** | **Impact of  case-mix** |
| --- | --- | --- | --- | --- | --- | --- | --- | --- | --- | --- | --- | --- | --- | --- | --- |
| **De Graaff** | 2023 | Netherlands | Liver metastases | Secondary aim assess hospital variation in 5 year OS after liver metastases surgery | Population-based study. Hospital-level and network- level comparison. | Multilevel, Cox proportional hazards modelling (Cox PH) | N=2,820 diagnosed between 2014 and 2017. | 5-year OS | | - **Basic demographics:**  Sex, age - **Patient factors:** Body mass index (BMI), American Society of Anaesthesiologists (ASA) grade, and Charlson Comorbidity Index (CCI). - **Tumour factors:** Number of metastasis, diameter of the largest metastasis, synchronous or metachronous metastases, CEA, pathological N-stage of primary tumour, location of primary tumour (rectal/colon), bilobar disease, and extrahepatic disease. - **Treatment factors**: surgical procedure (open/ laparoscopic), major liver resection - **Other factor**:  Type of hospital | | No model description. No internal or external validation. | | Not reported. | They presented volume funnel plots of both un-adjusted and adjusted 5-year overall survival. |
| **Kowalski** | 2022 | Germany | Colorectal cancer | To compare Quality of life aspects in patients undergoing colorectal surgery | Prospective study. Hospital-level comparison | Multilevel, multivariable linear regression | N=3,142 included between October 2018 and December 2019 | Quality of life (QoL) | | - **Basic demographics:**  Sex, age - **Patient factors:**  Baseline scores on EORTC QLQ-C30 and -CR29, ASA score - **Tumor factors:** stage, site (colon/rectum), and presence of a stoma after 12 months. - **Socioeconomic factors:** nationality, education level, health insurance status | | Model described. Internal validation by 10-fold cross validation | | **Discrimination**: R^2^ for the different models ranged between 0.12 to 0.45. The strongest model was for physical functioning | They presented crude and adjusted scores at 12-month follow-up displaying observed to expected (O/E)-ratios in a volume funnel plot |
| **Author** | **Year** | **Country** | **Cancer** | **Aim** | **Study design** | **Modelling method** | **Number of cases** | **Outcome** | | **Case-mix factors** | | **Validation** | | **Performance metrics** | **Impact of  case-mix** |
| **Nilssen** | 2024 | Norway | Small cell lung cancer | Compare OS between healthcare regions in limited disease of small cell lung cancer | Population-based study. Regional-level comparison | Kaplan-Meier and multilevel Cox PH | N =660  diagnosed 2018-2022 | OS | | - **Basic demographics:**  Sex, age - **Patient factors:**  Performance status (WHO/ECOG). - **Tumor factors:**  Stage | | Model described. No internal or external validation. | | Not reported | They showed Kaplan Meyer curves and Cox PH curves to visualize the crude- and adjusted mortality rates |
| **Thurell** | 2023 | Sweden | Breast cancer | Compare overall survival between healthcare regions | Population-based study. Regional-level comparison | Cox PH | N=2,654 diagnosed between 2008-2018 | | 5-year OS and 10-year OS | | - **Basic demographics:**  Age - **Patient factors:** Menopausal status - **Tumor factors:**  Tumor stage, nodal stage, histological grade, estrogen receptor status, progesterone receptor status, age | | Model described. No internal or external validation | Not reported | They presented crude survival curves and Cox PH curves to visualize the impact of case-mix adjustment |
| **Govaert** | 2016 | Netherlands | Colorectal cancer | Compare complication rates between hospitals and relate these to costs | Population-based study. Hospital-level comparison | Multivariable logistic regression | N=9,913 diagnosed between 2010-2012 | | 30-day severe complication or mortality | | - **Basic demographics:**  Sex, age - **Patient factors:** BMI, CCI, ASA grade - **Tumor factors:** Presence of double tumor, location of tumor, stage of tumor (TNM), urgency of resection - **Treatment factors:**  Preoperative radiotherapy, resection of distant metastases, and urgency of resection | | No model description. No internal or external validation | Not reported | Not reported |
| **Gilthorpe** | 2011 | UK | Colorectal cancer | Exploring and illustrating how multilevel latent class modelling could be applied for risk adjustment | Population-based study. Hospital-level comparison | Multilevel latent class modelling, nesting patients within Trusts. | N =24,640 diagnosed between 1998-2004 | | 3-year all-cause mortality | | - **Basic demographics:** Age, sex - **Tumor factors:** tumor stage (Dukes classification) - **Socioeconomic factors:** Socioeconomic background | | No model description. Internal validation (boot-strapping) | Not reported | Not reported |
| **Author** | **Year** | **Country** | **Cancer** | **Aim** | **Study design** | **Modelling method** | **Number of cases** | | **Outcome** | | **Case-mix factors** | | **Validation** | **Performance metrics** | **Impact of  case-mix** |
| **Algera** | 2023, July | Netherlands | Advanced-stage ovarian cancer | Developing a risk-adjusted textbook outcome for cytoreductive surgery in ovarian cancer. | Ppopulation-based study. Hospital-level comparison. | Logistic regression | N=1,909 diagnosed between 2017-2020. | | Textbook outcome | | - **Basic demographics:**  Age - **Patient factors:**   performance status (WHO/ECOG), comorbidity burden (Charlson Comorbidity index), BMI - **Tumor factors:**  Stage, tumor histology - **Other factors:** previous abdominal surgery | | Model described. No internal or external validation | Not reported  . | Crude- and adjusted volume funnel plots were used |
| **Ten Berge** | 2021 | Netherlands | Non-small cell lung cancer | Developing and testing if textbook outcome can improve quality of surgical care | Population-based study. Hospital-level comparison. | Logistic regression | N=5,513 | | Textbook outcome | | - **Basic demographics:**  Sex, age - **Patient factors:**  ASA class, comorbidities - **Tumor factors:**   Clinical stage   - **Other factors:**   Previous thoracic surgery | | Model described. No internal or external validation. | Not reported. | Adjusted volume funnel plots were shown. However, no un-adjusted funnel plots. |
| **Tighe** | 2023 | UK | Facial, non-melanoma skin cancer | Developing a risk-adjustment model for postoperative positive resection margins after resection of non-melanoma skin cancer in the head- and neck region | Retrospective cohort study with manually extracted data from three hospitals’ electronic health records (EHR).  Hospital-level comparison | Machine learning (Auto-Sklearn) | N=3,354  diagnosed between January 2017 and January 2020 | | Models for basal cell cancer (BCC) and squamous cell carcinoma (SCC) | | - **Basic demographics:** Age, gender - **Tumor factors:** Tumor dimension, tumor thickness and level of invasion, perineural and lymphovascular invasion as well as grade were also used as candidate predictors for SCC. Histological subtype was also used for BCC | | No model description. Internal validation using 10-fold cross-validation. | Sensitivity (recall), precision (positive predictive value), AUC.  AUC=0.71for SCC and 0.67 for BCC | Volume funnel plot comparing crude- and adjusted positive margin rate |
| **Fischer** | 2016 | UK | Gastro-esophageal cancer | Develop case-mix adjustment models for the described outcomes | Population-based retrospective study. Hospital-level comparison | Logistic regression | N=4,882 patients that underwent surgery for gastro-esophageal cancer April 2011 and March 2013 | | 30- and 90 day mortality rates and anasto-motic leakage rates | | - **Basic demographics:**  Sex, age - **Patient factors**  Comorbidity count, ECOG, ASA class - **Tumor factors:**  T-stage, N-stage, histology of the tumor, tumor location - **Socioeconomic factors:** Social deprivation | | Model described. Internal validation using bootstrapping. | **Discrimination:** AUC ranging between 0.59-0.66 for the three models. **Calibration:** Scatter plot of observed to expected outcomes | Not reported |
| **Author** | **Year** | **Country** | **Cancer** | **Aim** | **Study design** | **Modelling method** | **Number of cases** | | **Outcome** | | **Case-mix factors** | | **Validation** | **Performance metrics** | **Impact of  case-mix** |
| **Damhuis** | 2006 | Netherlands and UK | Lung cancer | To develop a case-mix model for 30-day postoperative mortality | Population-based study. Regional-level comparison. | Logistic regression | N=6,110 operated for lung cancer between 1993-2002 | | 30-day post-operative mortality | | - **Basic demographics:** Age, gender, - **Tumor factors:**  Histological subtype. Stage (TNM) was not a significant predictor when type of surgery was included. - **Treatment factors:** Type of surgery | | Model described. No internal or external validation | **Discrimination:** AUC 0.71.  **Calibration:** Hosmer Lemenshow statistics p=0.68, indicating good calibration | Not reported |
| **Kolfschoten** | 2013 | Netherlands | Colon cancer | To assess between-hospital variations in achieving a textbook outcome after colon cancer resection. | Population-based study. Hospital-level comparison. | Logistic regression | N=5,582 from 82 Dutch hospitals operated for colon cancer in year 2010. | | Textbook outcome | | - **Basic demographics:**  Sex, age - **Patient factors**: BMI, CCI, ASA - **Tumor factors**:  Tumor stage, urgency - **Treatment factors:** Operative procedures and additional resection for other tumor or metastasis. Tumor complications - **Other:**  Tumor complications | | Model described. No internal or external validation | **Discrimination:** AUC=0.75 | Funnel plot for un-adjusted textbook outcome variations. Observed/Expected (O/E) hospital rankings for adjusted outcomes |
| **Boyle** | 2023 | UK | Colo-rectal cancer | To assess hospital variation in acute toxicity after systemic anti-cancer therapy (SACT) | Population-based study. Hospital-level comparison. | Logistic regression | Patients were april 2016 and march 2019 at 106 UK hospitals  Stage III-model: N=8,173  Stage IV-model: N=7,683 | | A composite indicator summarizing several acute toxicities from SACT | | - **Basic demographics:**  Sex, age - **Patient factors:**  Comorbidity burden, performance status - **Tumor factors:**  Tumor site, staging - **Socioeconomic factors:** Socioeconomic status | | Model described. No internal or external validation. | Discrimination: AUC ranged from 0.580.64.  **Calibration:** Hosmer-Lemenshow test: p=0.71 and 0.90 for the two models. | Un-adjusted and adjusted volume funnel plots were used to visualize hospital variation in toxicity after SACT. |
| **Author** | **Year** | **Country** | **Cancer** | **Aim** | **Study design** | **Modelling method** | **Number of cases** | | **Outcome** | | **Case-mix factors** | | **Validation** | **Performance metrics** | **Impact of  case-mix** |
| **Tighe** | 2021 | UK | Squamous cell cancer in the head and neck region | To develop a case-mix adjustment model for positive surgical margin rates | Retrospective cohort study with manually extracted data from three hospitals’ electronic health records (EHR) | Compared four different machine learning approaches:  1. J48 (decision tree)  2. Random forest  3. Naïve-Bayes classifier.  4. Logistic regrrssion. | N=1,316 patients | | Positive surgical margin rate. | | - **Basic demographics:**  Sex, age - **Patient factors**  Comorbidity burden (ACE-27), performance status (WHO/ECOG) - **Tumor factors:**  Stage - **Treatment factors:**  Operative treatment and anesthetic treatment, case complexity (using the BUPA severity of surgery index) | | No model description. Internal validation using 10-fold cross-validation. | **Discrimination:** AUC varied between 0.573-0.72.  **Calibration (only champion model):**  Hosmer Lemeshow test p=0.90 | Adjusted volume funnel plot was presented but no un-adjusted version for comparison |
| **Talsma** | 2014 | Netherlands | Esophageal cancer | Third aim was to develop a case-mix adjustment model to compare postoperative complication rates between hospitals | Single-center retrospective cohort study | Logistic regression | N=1,282 patients operated between 1991 and October 2011 at Erasmus MC | | 30-day and 90-day postoperative mortality rates | | - **Basic demographics:**  Sex, age - **Patient factors:** Cardiovascular disease, pulmonary disease and pre-operative weight loss - **Treatment factors:** Surgical approach, resection margin | | Model described. No internal or external validation | Not reported | Not reported |
| **Author** | **Year** | **Country** | **Cancer** | **Aim** | **Study design** | **Modelling method** | **Number of cases** | | **Outcome** | | **Case-mix factors** | | **Validation** | **Performance metrics** | **Impact of  case-mix** |
| **Walker** | 2015 | UK | Colorectal cancer | To develop and validate a risk-adjustment model for 90-day postoperative mortality | Population-based study. Hospital-network (Trusts) level comparison.  Registry-study. | Logistic regression | N=62,314 | | 90-day postoperative mortality | | - **Basic demographics:**  Sex, age - **Patient factors:** ASA grade, comorbidity burden - **Tumor factors:**  Tumor status, Nodal status, Metastasis status, mode of admission, cancer site - **Other factors:**  Audit year | | Model described. Internal validation using bootstrapping. | **Discrimination:** AUC=0.80  **Calibration:** Comparing observed to expected outcomes in deciles of risk. Showed no significant differences | Not reported |
| **Tighe** | 2017, June | UK | Squamous cell cancer in the head and neck region | To validate a previously developed case-mix adjustment model | Case audits, some data retrospective and some prospective. Hospital-level comparison. | Logistic regression | N=188 patients operated for head neck cancer at a surgical unit between June 2013 and June 2015. | | Postoperative morbidity as a composite of several complications. | | - **Basic demographics** Age - **Patient factors:**  WHO performance status - **Treatment factors:**  Surgical complexity (according to the BUPA classification) - **Other:**  Intraoperative blood loss. | | External validation | **Discrimination:** AUC=0.70 compared with 0.76 in the development set **Calibration:** Hosmer-Lemeshow test on deciles of risk p=0.48  Overall accuracy: Brier score=0.35 indicating problematic overall performance | They present both crude and adjusted morbidity rates in a volume funnel plot. |
| **McArdle** | 2002 | UK | Colo-rectal cancer | To assess if case-mix adjustment had an impact on survival benchmarking in colorectal cancer. | Retrospective multicenter study where data was extracted from case notes. Hospital-level comparison | Cox PH | N=3200 | | 5-year OS and cancer-specific survival | | - **Basic demographics:**  Age, sex - **Tumor factors:**  Presentation (emergency surgery or elective surgery) and Duke’s stage. - **Socioeconomic factors:** Socioeconomic status | | No model description. No internal or external validation | Not reported | Presented crude- versus adjusted survival rates for each hospital to show how case-mix factors influenced the survival estimates. |
| **Author** | **Year** | **Country** | **Cancer** | **Aim** | **Study design** | **Modelling method** | **Number of cases** | | **Outcome** | | **Case-mix factors** | | **Validation** | **Performance metrics** | **Impact of  case-mix** |
| **Matthes-Martin** | 2008 | Germany and Austria | Pediatric hematological malignancies | To develop a risk-adjustment model predicting 1-year survival after allogenic stem cell transplantation | Registry-based retrospective multicenter study. Hospital-level comparison | Logistic regression and mixed effects Logistic regression | N=1291 | | 1-year treatment-related mortality (TRM) rates were compared | | - **Basic demographics:**  Age - **Patient factors:**  Prior stem cell transplantation - **Tumor factors:**  Hematopoietic malignancy, disease stage, unrelated/mismatched donor, and type of graft | | Model described. Internal validation (cross-validation by dividing the dataset into five parts and assessing model in each subset. | **Discrimination**: AUC=0.674  **Calibration:** Hosmer Lemeshow test was performed but not reported | They displayed observed to expected 1-year TRM for all included hospitals |
| **Talsma** | 2011 | Netherlands | Breast cancer | To develop a case-mix adjustment model for re-resection rates after breast conserving surgery. | Registry-based multicenter study. Hospital-level comparison. | Logistic regression | N=1,923 patients operated in 2006 or 2007 in one of 15 Dutch hospitals. | | Re-resection rates after breast conserving surgery. | | - **Basic demographics**  Age - **Tumor factors:**  Histology, tumor location, histological grading, T-stage, N-stage, multifocality, hormonal receptor status and pre-operative diagnosis | | Model described. No internal or external validation. | **Discrimination:** AUC=0.73  **Calibration:** Hosmer-Lemeshow test p=0.31. | Not reported |
| **Beck** | 2018 | Netherlands | Lung cancer | Develop a risk-adjustment model predicting 30-day postoperative mortality rates after lung resections for non-small cell lung cancer. | Registry-based retrospective multicenter study. Hospital-level comparison | Logistic regression | N=6,600 from 37 hospitals | | 30-day postoperative mortality rates | | - **Basic demographics:**  Sex, age - **Patient factors:**  ECOG, ASA, cardiac morbidity, lung function - **Tumor factors:** Primary or recurrent tumor - **Treatment factors:** Induction therapy and extent of surgery | | Model described. No internal or external validation. | **Discrimination:** AUC=0.81 | Not reported |
| **Author** | **Year** | **Country** | **Cancer** | **Aim** | **Study design** | **Modelling method** | **Number of cases** | | **Outcome** | | **Case-mix factors** | | **Validation** | **Performance metrics** | **Impact of  case-mix** |
| **Elfrink** | 2021, oct | Netherlands | Liver metastases from colorectal cancer | Describe case-mix differences between the seven oncological hospital networks in the Netherlands and assess adjusted postoperative survival disparities, | Registry-based retrospective multicenter study. Hospital-network level comparison | Logistic regression | N=5,383 patients that underwent liver resection between 2014 and 2019 from 26 hospitals in 7 oncological networks | | 30-day major morbidity and 30-day mortality rates | | - **Basic demographics:**  Sex, age - **Patient factors:**  ASA, BMI, CCI, previous liver surgery - **Tumor factors:**  Histology, number of liver metastases, diameter of largest metastasis - **Treatment factors:**  Preoperative chemotherapy, thermal ablation, minor or major resection **Other:**  Type of hospital | | Model described. No internal or external validation | Not reported | They presented un-adjusted and adjusted volume funnel plots with 95 % confidence interval |
| **Bernard** | 2011 | France | Non small cell lung cancer | Develop and validate a risk-adjsutment model for 30-day postoperative mortality after lung cancer resection | Registry-based retrospective multi-centre study. Hospital-level comparison | Logistic regression | N=19,049 patients from 81 hospitals undergoing lung resection between 2003 and 2008. | | 30-day post-operative mortality | | - **Basic demographics:**  Sex, age - **Patient factors** ASA-score, lung function, ECOG, BMI, prior thoracic surgery, smoking status and comorbidity burden - **Tumor factors:**  Side of resection, tumor stage - **Treatment factors:**  Type of resection, surgical margins, use of steroids | | Model described. Internal validation using bootstrapping | **Discrimination:** AUC=0.78  **Calibration:** Calibration plot showed a slop near 1, indicating good calibration | Not reported |
| **Nickelsen** | 2005 | Denmark | Colorectal adenocarcinoma | To compare 30-day postoperative mortality between Danish hospitals both un-adjusted and adjusted for case mix. | Registry-based retrospective multi-centre study. Hospital-level comparison | Logistic regression | N=5,138 patients from 44 Danish surgical departments diagnosed between May 2001 and December 2002. | | 30-day post-operative mortality | | - **Basic demographics:**  Sex, age - **Patient factors:** ASA - **Tumor factors:**  Duke’s classification, tumor location, urgency | | Model described. No internal or external validation | Not reported | They compared un-adjusted to adjusted survival rates |
| **Author** | **Year** | **Country** | **Cancer** | **Aim** | **Study design** | **Modelling method** | **Number of cases** | | **Outcome** | | **Case-mix factors** | | **Validation** | **Performance metrics** | **Impact of  case-mix** |
| **Algera** | 2023, April | Netherlands | Ovarian cancer | To develop a case-mix adjustment model for complicate course after cytoreductive surgery of ovarian cancer | Population-based, retrospective registry study. Hospital-level comparison | Logistic regression | N=1,822 patients who underwent cytoreductive surgery for ovarian cancer between 1st Jan 2017 and 31^st^ Dec 2019 in 21 Dutch hospitals | | Complicated course post-surgery (composite measure) | | - **Basic demographics:**  Age - **Patient factors:**   WHO performance status, BMI, CCI, previous abdominal surgery - **Tumor factors:** FIGO stage, histology **Treatment factors:**  Type of surgery (primary versus interval) and the outcome indicator complete versus incomplete cytoreduction | | Model described. No internal or external validation | Not reported | They compared un-adjusted to adjusted results in separate volume funnel plots with 95 % confidence interval |
| **Salet** | 2023 | Netherlands | Colon cancer | Compare variation in several short-term outcomes after laparoscopic colorectal surgery. A secondary aim was to assess the impact of hospital, surgeon, chance and case-mix on outcomes. | Population-based, retrospective registry study. Hospital- and surgeon-level comparison. | Logistic regression for binary outcomes and linear regression for continuous outcomes. Multilevel (hospital, surgeon, patient), mixed effects modelling. | N=6,640 patients from 48 Dutch hospitals (181 surgeons) that underwent colorectal laparoscopic surgery between 1^st^ of Jan 2018 and 31^st^ of Dec 2019. | | In-hospital mortality, 30-day readmission, LOS and more. | | - **Basic demographics:**  Age, sex - **Patient factors:**  Comorbidity burden (Elixhauser index). - **Socioeconomic factors:** Socioeconomic status | | No model description. No internal or external validation | Not reported | They used variance partition calculation (VPC) to assess the impact of case-mix in each outcome |
| **Author** | **Year** | **Country** | **Cancer** | **Aim** | **Study design** | **Modelling method** | **Number of cases** | | **Outcome** | | **Case-mix factors** | | **Validation** | **Performance metrics** | **Impact of  case-mix** |
| **Van der Heiden** | 2012 | Netherlands | Breast cancer | Compare positive surgical margin rates after breast-conserving surgery between Dutch hospitals adjusted for case mix | Population-based study. Hospital-level comparison | Logistic regression | N=7,146 patients from 96 Dutch hospitals, undergoing surgery between July 2008 and June 2009 | | Postoperative positive margins | | - **Basic demographics:**  Age - **Tumor factors:**  Tumor size, multifocality, nodal status, histological subtype, and histological grade | | Model described. No internal or external validation | Not reported | They compared un-adjusted to adjusted survival rates but as there were 95 hospitals they did not show how each individual hospital was affected by case mix |
| **Busweiler** | 2017 | Netherlands | Gastro-esophageal cancer | Construct and analyze between-hospital variation in a composite measurement of quality | Registry-based retrospective multicenter study. Hospital-level comparison | Logistic regression | N=4,520 patients that underwent surgery at Dutch hospitals between January 2011 and December 2014 | | Textbook outcome | | - **Basic demographics:**  Sex, age - **Patient factors:**  BMI, ASA-grade, CCI, previous surgery - **Tumor factors:**  Tumor stage, tumor location, urgency - **Treatment factors:**  Type of surgery, surgical approach, and additional resection due to invasiveness | | Model described. No internal or external validation | Not reported | Not reported |
| **Author** | **Year** | **Country** | **Cancer** | **Aim** | **Study design** | **Modelling method** | **Number of cases** | | **Outcome** | | **Case-mix factors** | | **Validation** | **Performance metrics** | **Impact of  case-mix** |
| **Voeten** | 2020 | Netherlands | Esophageal cancer | To explore the incidence of failure to cure and assess hospital variations in rates of failure to cure | Registry-based retrospective multicenter study. Hospital-level comparison | Logistic regression | N=5,894 patients operated for esophageal cancer between 2011 and 2018 from 22 hospitals. | | Rates of “Failure to cure” | | - **Basic demographics:**  Sex, age - **Patient factors:**  Preoperative loss in kilograms, ASA score, BMI, CCI, previous upper gastrointestinal surgery - **Tumor factors:** Clinical T-stage, clinical N-stage, TNM-stage, tumor location - **Treatment factors:** Surgical procedure, neoadjuvant chemotherapy - **Other:** Year of resection and , annual surgical volume | | Model described. No internal or external validation | Not reported | Not reported |
| **Snijders** | 2013 | Netherlands | Colorectal cancer | To explore how rates of anastomotic leakage after colorectal surgery is affected by case-mix and treatment factors. Secondly, they aimed to compare case-mix-adjusted mortality rates | Population-based retrospective multicenter study. Hospital-level comparison | Logistic regression | N=15,236 patients from 83 hospitals operated for colorectal cancer between 1^st^ January 2009 and 31^st^ of December 2011. | | Anasto-motic leakage and postoperative mortality | | - **Basic demographics:**  Sex, age - **Patient factors:**  ASA-score, BMI, comorbidity burden, steroid treatment, previous abdominal surgery, weight loss, nutritional status, alcohol abuse, smoking, leukocytosis. - **Tumor factors:**  Tumor stage, tumor location and, urgency. - **Treatment factors:** Additional resection | | Model described. No internal or external validation | Not reported | They used three models: 1. Un-adjusted model, 2. Adjusted for case-mix and 3. Adjusted for both case-mix and treatment factors. They then showed how each hospital ranked in the outcome according to model 1 and displayed how the ranking changed using model 2 and 3. |
| **Author** | **Year** | **Country** | **Cancer** | **Aim** | **Study design** | **Modelling method** | **Number of cases** | | **Outcome** | | **Case-mix factors** | | **Validation** | **Performance metrics** | **Impact of  case-mix** |
| **Tighe** | 2019, Oct | UK | Squamous cell cancer in the head and neck region | To apply machine learning methods to develop case-mix adjustment models for complication rates | Case audits, some data retrospective and some prospective. Hospital-level comparison | J48 decision trees separately, random forests separately and both of these integrated in the more complex Auto-WEKA machine learning environment. | N=1,254 patients who underwent surgery at 6 UK hospitals. | | They developed four models:  1. 30-day post-operative compli-cations  2. 30-day severe post-operative compli-cations  3. Length of stay  4. 30-day compli-cation rates in patients receiving free tissue transfer | | The candidate variables included:   - **Basic demographics:**  Sex, age - **Patient factors:** Alcohol use, performance status, smoking, comorbidity burden, tracheostomy use, previous radiotherapy and, previous surgery. - **Tumor factors:**  Scale of operation, high risk operation, T-stage, N-stage. | | No model description. Internal validation using 10-fold cross-validation. | **Discrimination:** AUC ranged from 0.549 to 0.824 between the different models and outcomes | Not reported |
| **Morris** | 2011 | UK | Colorectal cancer | To compare 30-day postoperative mortality rates between the NHS hospital trusts | Registry-based retrospective multicenter study. Hospital-network-level comparison | Mixed effects logistic regression | N=160,920 patients that underwent colorectal surgery for cancer between 1998 and 2006 | | 30-day post-operative mortality | | - **Basic demographics:**  Sex, age - **Patient factors:** CCI - **Tumor factors:** emergency operation, Duke’s stage, cancer site - **Socioeconomic factors:** Postal code income - **Other factors:** Calendar year of diagnosis | | Model described. No internal or external validation | Not reported | Un-adjusted versus case-mix adjusted volume-funnel plots showed the impact of case-mix |
| **Author** | **Year** | **Country** | **Cancer** | **Aim** | **Study design** | **Modelling method** | **Number of cases** | | **Outcome** | | **Case-mix factors** | | **Validation** | **Performance metrics** | **Impact of  case-mix** |
| **Elfrink** | 2021, oct | Netherlands | Colorectal liver metastasis | Develop and validate a case-mix adjustment model for postoperative mortality and severe morbidity | Registry-based retrospective multicenter study. Hospital- level comparison | Logistic regression | Development cohort: N=4,639 patients who underwent surgery for colorectal liver metastases between 2014 and 2018 from Dutch hospitals. Validation cohort: 778 patients who underwent the same surgery in 2019 in 23 Dutch hospitals | | 30-day post-operative mortality and 30-day severe co-morbidity rate (Clavien-Dindo 3a or higher) | | - **Basic demographics:**  Sex, age - **Patient factors:** CCI, BMI, ASA, previous liver resection - **Tumor factors:** Histopathological liver parenchyma, number of tumor lesions, largest metastasis diameter, location of primary tumor, timing of metastasis, extrahepatic disease. - **Treatment factors:** Preoperative chemotherapy, major liver resection, bi-lobar disease - **Other:**  Type of hospital. | | Model described. Internal validation by dividing the whole cohort in a development set and a validation set. | Not reported. | They show the O/E-ratio in a volume funnel plot with 95 % limits for confidence interval. |
| **Henneman** | 2014 | Netherlands | Colorectal cancer | To assess the impact of case mix and random variation when comparing postoperative mortality rates between Dutch hospitals | Registry-based retrospective multicenter study. Hospital-level comparison | Logistic regression with fixed effects and with random effects. | N=25,591 patients that underwent colorectal surgery between 1^st^ Jan 2009 and 31^st^ Dec 2011 in 92 Dutch hospitals | | 30-day post-operative mortality | | - **Basic demographics:**  Sex, age - **Patient factors:** ASA, CCI, BMI - **Tumor factors:**  TNM stage, tumor location, preoperative tumor condition, urgent operation, and synchronous colorectal tumors. - **Treatment factors:** Procedure, preoperative (chemo)-radiotherapy, and additional resections | | No model description. No internal or external validation. | Not reported. | They provided un-adjusted hospital rankings versus adjusted with a fixed effects model versus adjusted with a random effects model. They also controlled how rankings for individual hospitals changed with different models. |
| **Author** | **Year** | **Country** | **Cancer** | **Aim** | **Study design** | **Modelling method** | **Number of cases** | | **Outcome** | | **Case-mix factors** | | **Validation** | **Performance metrics** | **Impact of  case-mix** |
| **Tighe** | 2019, Nov, LOS | UK | Head and neck cancer | To build a case-mix adjustment model for postoperative hospital length of stay | Case audits from 5 UK hospitals | Linear regression and decision tree analyses (Machine learning) | Development set: N=638 patients that underwent surgery for head and neck cancer between 2009 and 2015. External validation set: N=112 patients from a fifth UK hospital during the same time period. | | Post-operative length of stay in operating hospital | | - **Basic demographics:**  Sex, age - **Patient factors:** Alcohol usage, smoking, comorbidity index, performance status, - **Tumor factors:**  High risk operation, T-class, N-class - **Treatment factors:** Free flap, tracheostomy, scale of operation, and previous radiotherapy. | | No model description. Internal validation (splitting the training set into a development set (60 %) and validation set (40 %).  External validation: Data from another hospital. | **Discrimination:** They report sensitivity and specificity for the decision tree model. Adjusted R^2^ for the linear model. | They compared frequencies of longer than expected, expected and shorter than expected LOS at different hospitals in a histogram. |
| **Tighe** | 2019, Nov, validation | UK | Squamous cell cancer in the head and neck region | To validate a case mix adjustment model for postoperative morbidity | Case audits, some data retrospective and some prospective. Hospital-level comparison | Machine learning (Neural networks) | N=195 patients that underwent surgery between 2015-2017 at two UK hospitals | | Post-operative morbidity defined as any comp-lication of any degree within 30 days of head and neck surgery | | - **Basic demographics:**  Sex, age - **Patient factors:** Comorbidity index, malnutrition status, ECOG status, - **Tumor factors:** TNM-classification - **Treatment factors:** Operative- and anesthetic treatments | | No model description. External validation | **Discrimination:** ROC curve and AUC.  **Calibration:** Calibration plot and overall observed to expected complications | Visualized un-adjusted versus adjusted complication rates in a volume funnel plot |
| **Author** | **Year** | **Country** | **Cancer** | **Aim** | **Study design** | **Modelling method** | **Number of cases** | | **Outcome** | | **Case-mix factors** | | **Validation** | **Performance metrics** | **Impact of  case-mix** |
| **Tighe** | 2014, Dec | UK | Squamous cell cancer in the head and neck region | To assess the impact of different case mix factors on morbidity rates and mortality rates after surgery. A secondary aim was to develop case-mix adjustment models for both outcomes | Registry-based retrospective single center study. Hospital-level comparison | Logistic regression | N=396 patients from one operating surgeon who underwent surgery between 1992 and 2010 | | 30-day post-operative mortality and 30-day post-operative comp-lication rates. | | - **Basic demographics:**  Sex, age - **Patient factors:** Smoking status, alcohol consumption, ECOG score, ASA-score, MUST score, comorbidity burden, abdominal history, other history, - **Tumor factors:** T-stage, N-stage, and extracapsular spread. - **Treatment factors:** Use of flap, tracheostomy, anesthetic time, and scale of operation. - **Other:** Estimated intra-operative blood loss, and surgical margins | | Model described. No internal or external validation. | **Discrimination:** ROC-curve and AUC. | Not reported |
| **Kolfschoten** | 2011 | Netherlands | Colorectal cancer | Assess how case-mix factors influence postoperative 30-day mortality rates on hospital level and in relation to hospital volume and teaching status | Registry-based retrospective multicenter study. Hospital- level comparison. | Logistic regression | N=8,580 patients who underwent surgery for colorectal cancer in 2010 at one of 90 Dutch hospitals. | | 30-day post-operative mortality | | - **Basic demographics:**  Sex, age - **Patient factors:** Comorbidity burden, ASA-class, BMI - **Tumor factors:** TNM-stage, , preoperative tumor complications, urgency of surgery, - **Treatment factors:** type of resection, additional resection for metastases/non-radicality, and neoadjuvant (chemo)-radiation | | Model described. No internal or external validation. | **Discrimination:** C-statistic:  Colon: 0.81 (0.78-0.83) and Rectum: 0.87 (0.82-0.91) | Not reported |
| **Jack** | 2003 | UL | Lung cancer | Assess hospital variations in 1- and 3-year survival rates for lung cancer | Registry-based retrospective, multicenter study. Hospital level comparison | Mixed effects, logistic regression | N=32,818 patients who underwent treatment for lung cancer between 1995 and 1999 from 26 UK hospitals | | 1- and 3-year survival. | | - **Patient demographics:** Sex, and age - **Tumor factors:** Disease stage, histological type, basis of diagnosis - **Socioeconomic factors:** socioeconomic status - **Treatment factors:**  Treatment variables (unspecified) | | No model description. No internal or external validation. | Not reported | Not reported |
| **Author** | **Year** | **Country** | **Cancer** | **Aim** | **Study design** | **Modelling method** | **Number of cases** | | **Outcome** | | **Case-mix factors** | | **Validation** | **Performance metrics** | **Impact of  case-mix** |
| **Fischer** | 2015 | Netherlands | Colon cancer | Evaluate how case-mix and random variation from low hospital case volumes (i.e. noise) impact the two studied outcomes | Population-based study. Hospital-level comparison. | Fixed effects logistic regression to assess the impact of case mix and random effects logistic regression to assess the impact of noise | N=13,120 patients from 85 Dutch hospitals diagnosed between 2011-2012 | | Anasto-motic leakage (AL) and post-operative mortality | | - **Basic demographics:**  Sex, age - **Patient factors:** CCI, ASA score - **Tumor factors:**  Operation urgency, , histological type, stage (TNM-classification), preoperative complication and number of- and location of tumor. - **Other:** Year of operation | | Model described. No internal or external validation | **Discrimination:** AUC | Plotted standardized ratios for the random effects models (Y-axis) against the crude and case-mix adjusted standardized ratios separately |
| **Tighe** | 2014, sept | UK | Squamous cell cancer in the head and neck region | To develop and validate a case mix adjustment model for postoperative complication rates. | Case audits, some data retrospective and some prospective. Hospital-level compariso | Logistic regression | N=901 patients who underwent surgery at 3 UK hospitals between 2009 and 2012 | | Post-operative morbidity defined as any comp-lication of any degree within 30 days of head and neck surgery | | - **Basic demographics:**  Sex, age - **Patient factors:** Smoking status, alcohol consumption, ECOG score, ASA-score, MUST score, comorbidity burden, abdominal history, and other history - **Tumor factors:** T-stage, N-stage, and extracapsular spread - **Treatment factors:** Anesthetic time, use of flap, tracheostomy, scale of operation - **Other factors:** Surgical margins, and estimated intra-operative blood loss | | Model described. Internal validation through data splitting into a development set (70 %) and a validation set (30 %). No external validation. | **Discrimination:** ROC-curve and AUC. | Displayed a volume funnel plot showing both crude and adjusted complication rates for all three sites. |
| **Das** | 2006 | UK | Gynecological cancers | To assess if the P-POSSUM model could be applied for risk-adjustment in gyno-oncological surgery | Case audits of all patients who underwent surgery for a gynecological malignancy at one hospital. | Logistic regression | N=468 patients who underwent surgery for a gyne-cological malignancy at one UK center between September 2002 and August 2003 | | 30-day postoperative mortality | | Parameters in the P-POSSUM (18 variables, 12 related to the patient and 6 related to the complexity of surgery) where operative severity was adapted to work for gynecological cancer surgery. | | Model described External validation | **Calibration:** Comparing observed to expected mortality rates in quarters of risk. | Not reported. |
| **Author** | **Year** | **Country** | **Cancer** | **Aim** | **Study design** | **Modelling method** | **Number of cases** | | **Outcome** | | **Case-mix factors** | | **Validation** | **Performance metrics** | **Impact of  case-mix** |
| **Vos** | 2020, sept | Netherlands | Breast cancer | Assess how case-mix variation and random variation affected three outcome indicators and three process indicators applied in the Dutch breast cancer quality registry. | Registry-based retrospective multicenter study. Hospital-level comparison | Fixed effects logistic regression for case-mix effects, and random effects logistic regression for random effects | N=79,690 patients that underwent surgery for invasive breast cancer or ductal cancer in situ (DCIS) between January 2011 and August 2016 at 91 Dutch hospitals. | | Outcome indicators: Q1. Positive surgical margins after breast conserving surgery in breast cancer.  Q2. Positive surgical margins after breast conserving surgery for DCIS.  Q3. Rates of breast conserving surgery and direct reconstruction after mast-ectomy. | | - **Basic demographics:**  Sex, age - **Patient factors:**  ECOG score, previous breast surgery - **Tumor factors:**  Screen-detected, palpable tumor, multifocal, histology, histological grade, hormonal status, HER2-status, tumor size, pathological T-stage, pathological N-stage, and M-stage | | Model described. No internal or external validation. | **Discrimination:** AUC. | Impact of case-mix and random effects: Displaying a scatterplot for each indicator with the crude model on the y-axis and the adjusted model on the x-axis to see how case-mix and random effects makes the hospital scores deviate from the diagonal that would occur if the indicator was not at all affected by case-mix and random effects |
| **Vos2** | 2020, sept | Netherlands | Breast cancer | Develop a textbook outcome for breast cancer and compare case-mix adjusted rates of textbook outcomes | Registry-based retrospective multicenter study. Hospital-level comparison | Linear regression (continuous outcomes) and logistic regression (binary outcome). | N=79,690 patients underwent surgery for invasive breast cancer or ductal cancer in situ (DCIS) between January 2011 and August 2016 | | Textbook outcome | | - **Patient demographics:** Age - **Tumor factors:** Histology, pathological T-, and N- stage, differentiation grade, multifocality, and estrogen and Her2neu receptor status | | No model description. No internal or external validation. | Not reported. | They show how the interquartile range changes when removing case-mix effects. They also scatter plot the crude estimates compared with the adjusted estimates. |
| **Author** | **Year** | **Country** | **Cancer** | **Aim** | **Study design** | **Modelling method** | **Number of cases** | | **Outcome** | | **Case-mix factors** | | **Validation** | **Performance metrics** | **Impact of  case-mix** |
| **Tighe** | 2022 | UK | Squamous cell cancer in the head and neck region | To develop a risk-adjustment model for free-flap failure after surgery using machine learning methods | Case audits, some data retrospective and some prospective. Hospital-level comparison | Explored several complex machine learning methods including mainly methods from the families eXtreme Gradient Boosting (XGB) and Deep-Forest | N=1,624 patients from 8 UK hospitals who underwent head and neck surgery with free-flap transfer | | Free-flap failure | | - **Basic demographics:**  Sex, age - **Patient factors:** Alcohol use, smoking status, comorbidity burden, performance status, diabetes, peripheral vascular disease, previous radiotherapy, previous surgery, - **Tumor factors:** T-stage, N-stage - **Treatment factors:** High risk surgery, bilateral neck, composite flap, double flap, tracheostomy, radial forearm free flap, thigh flap, fibular flap, DCIA flap, scapular system, latissimus dorsi, rectus abdominis and other flap | | No model description. Internal validation using 10-fold cross-validation. No external validation. | **Discrimination**: AUC.  **Calibration (only champion model):**  Hosmer Lemeshow test and calibration plot. | Not reported. |
| **Tighe et al. Development** | 2017 | UK | Squamous cell cancer in the head and neck region | To compare the performance of four case mix adjustment methods on predicting postoperative morbidity | Case audits, some data retrospective and some prospective. Hospital-level comparison. | Logistic regression, decision tree, Bayes Classifier and Neural Network | N=979 patients that underwent surgery at 4 UK hospitals between 1993 and 2015 | | Post-operative morbidity defined as any comp-lication of any degree within 30 days of head and neck surgery | | - **Basic demographics:**  Sex, age - **Patient factors:** Comorbidity burden, malnutrition status, and ECOG score - **Tumor factors:**  TNM-classification - **Treatment factors:** Operative- and anastethic treatments. | | No model description. Internal validation by training and test set. No external validation. | **Discrimination:** ROC curve and AUC. The champion model was further tested for negative predictive value, positive predictive value, sensitivity and specificity. | Visualized un-adjusted versus adjusted complication rates in a volume funnel plot. |
| **Author** | **Year** | **Country** | **Cancer** | **Aim** | **Study design** | **Modelling method** | **Number of cases** | | **Outcome** | | **Case-mix factors** | | **Validation** | **Performance metrics** | **Impact of  case-mix** |
| **Sibert** | 2021 | Germany | Prostate cancer | To compare case-mix adjusted patient-reported outcomes 12 months after radical prostatectomy | Registry-based retrospective multicenter study. Hospital-level comparison | Linear regression | N=7,065 patients who underwent radical prostatectomy between July 2016 and July 2019 from 88 hospitals. | | 1-year mean adjusted PROMs | | - **Basic demographics:**  Age - **Patient factors:** Baseline EPIC-26 score for each domain, number of comorbidities - **Tumor factors:**  Risk classification of the tumor, - **Socioeconomic factors:** Educational level, health insurance type, nationality - **Treatment factors:**  Hormonal therapy before surgery, and active surveillance before surgery | | Model described. No internal or external validation | **Discrimination:** R^2^ | Un-adjusted versus adjusted results were compared using the Cohen’s d to appreciate the impact of case mix |
| **Wahba** | 2023 | UK | CNS-tumors | Develop a case-mix adjustment model for 30-day postoperative mortality after neurosurgical operations of CNS-tumors. They also developed a model for several types of neurosurgical operations to explore if indication-specific models are needed. | Registry-based retrospective multicenter study. Hospital-level comparison | Logistic regression | N=50,748 patients who underwent neurosurgery between April 2013 and March 2018 at UK hospitals | | 30-day post-operative mortality | | - **Basic demographics:**  Sex, age - **Patient factors:** Frailty and comorbidity burden. - **Tumor factors:** The overall model included details on the specific neurosurgical condition and emergency admission. - **Socioeconomic factors:** Socioeconomic deprivation | | Model described. No internal or external validation. | **Discrimination:** AUC  **Calibration:** Calibration plot | They use a caterpillar plot to show un-adjusted versus adjusted 30-day mortality for all neurosurgical units. |
| **Author** | **Year** | **Country** | **Cancer** | **Aim** | **Study design** | **Modelling method** | **Number of cases** | | **Outcome** | | **Case-mix factors** | | **Validation** | **Performance metrics** | **Impact of  case-mix** |
| **Elfrink** | 2021, April | Netherlands | Primary tumors of the liver and liver metastases. | To assess patient- and disease characteristics associated with “Failure to rescue” (FTR) and compare rates of FTR between hospitals | Registry-based retrospective multicenter study. Hospital- level and network-level comparison. | Logistic regression | N=4,961 patients who underwent liver surgery for primary or secondary tumor in Dutch hospitals between 2014 and 2017 | | 30-day post-operative FTR. FTR was defined as having severe post-operative comp-lication (at least 3a on the Clavien-Dindo scale) and then die from the comp-lication | | - **Basic demographics:**  Sex, age - **Patient factors:** BMI, CCI, ASA-score, and previous liver resection. - **Tumor factors:**  Tumor type, maximum diameter of largest tumor, histopathology **Treatment factors:** Preoperative chemotherapy, major liver resection, surgical approach, - **Other factors:** Type of hospital, and annual hospital volume. | | Model described, no internal or external validation. | Not reported. | They showed O/E rates in a volume-funnel plot with 95 % confidence interval. |
| **Gildea** | 2016 | UK | Endometrial cancer | To assess hospital- and cancer network variations in 30-day postoperative mortality rates before- and after case-mix adjustment. | Registry-based multicenter study. Hospital-level and cancer network level comparison. | Random effects logistic regression | N=38,332 women diagnosed with endometrial cancer in the UK between 2000 and 2009 | | 30-day post-operative mortality | | - **Basic demographics:** Age - **Tumor factors:** FIGO stage, and histological grade - **Socioeconomic factors:** Income deprivation - **Other factors:** Year of diagnosis | | Model described. No internal or external validation. | The authors describe that they tested calibration by using the Hosmer-Lemeshow test but do not display the results in the article. No other performance metrics are mentioned. | They use volume-funnel plots to compare crude- to adjusted hospital-level differences and cancer network differences. |
| **Author** | **Year** | **Country** | **Cancer** | **Aim** | **Study design** | **Modelling method** | **Number of cases** | | **Outcome** | | **Case-mix factors** | | **Validation** | **Performance metrics** | **Impact of  case-mix** |
| **Henneman** | 2013 | Netherlands | Colorectal cancer | To assess hospital variation in failure to rescue rates | Populaation-based retrospective registry-based study. Hospital-level comparison. | Logistic regression | N=24,667 patients that underwent surgery from 1^st^ Jan 2009 to 31^st^ Dec 2011 for colorectal cancer at 92 Dutch hospitals. | | 30-day failure to rescue (FTR). FTR was defined as getting a severe post-operative comp-lication and then die. | | No description of tested case-mix factors. The factors included in the model were:   - **Basic demographics:**  Sex, age - **Patient factors:** ASA score, CCI, - **Tumor factors:**  Emergency surgery, tumor location, preoperative tumor complications, and TNM-stage - **Treatment factors:** Neoadjuvant treatment | | No model description. No internal or external validation. | Not reported. | They use Volume funnel plots to show the O/E-ratios. |
| **Burnell** | 2016 | UK | Gynecological cancers | To assess hospital-variations in intraoperative and postoperative complication rates. More specifically they aimed to assess the impact of case-mix on studied outcomes. | Case audits. Hospital-level comparisons. | Logistic regression with Lasso. | N=2,948 patients that underwent surgery for gyne-cological cancers at ten UK hospitals between April 2010 and February 2012 | | Two outcomes: 1. Intra-operative comp-lication rates.  2. 30-day post-operative comp-lication rates. | | - **Basic demographics:**  Age - **Patient factors:** BMI, previous abdominal surgery, low albumin, several comorbidities, smoking, other malignancy, and ASA-class. - **Tumor factors:**  Type of cancer - **Treatment factors:** Laparoscopic approach, and surgical complexity - **Other factors:** Level of surgeon (consultant, sub-speciality trainee, trainee), duration of surgery, and estimated blood loss. | | Model described. Internal validation through leave-one-out cross-validation. No external validation. | **Discrimination:** AUC_Lasso_=0.663  **Calibration:** They plotted expected to observed complication rates in deciles of risk in a calibration plot (not showing the plot). They report the calibration slope=0.871 (0.717-1.068) | Volume funnel plot showing O/E-rates of all hospitals |
| **Author** | **Year** | **Country** | **Cancer** | **Aim** | **Study design** | **Modelling method** | **Number of cases** | | **Outcome** | | **Case-mix factors** | | **Validation** | **Performance metrics** | **Impact of  case-mix** |
| **De Graaff** | 2022 | Netherlands | Colorectal liver metastasis | Comparing case-mix adjusted rates of achieving textbook outcome | Registry-based retrospective multicenter study. Hospital- and network level comparison. | Logistic regression | N=1,711 patients who underwent liver resection between 2019 and 2020 at 23 Dutch hospitals in 7 cancer networks. | | Rates of achieving textbook outcome | | - **Basic demographics:**  Sex, age - **Patient factors:** CCI, ASA score, BMI, previous liver surgery - **Tumor factors:**  histological class of liver parenchyma, histopathology, number of lesions, maximal diameter of largest tumor, bi-lobar disease, extrahepatic disease, location of primary tumor, timing of metastasis - **Treatment factors:** Major liver resection, and preoperative chemotherapy - **Other factors:** Type of hospital, and annual hospital volume | | Model described. No internal or external validation | Not reported | Bar diagrams visualized the crude variations in achieving Textbook outcome and case mix adjusted volume funnel plots showed the adjusted variation |
| **Voeten** | 2021 | Netherlands | Esophageal cancer | To assess hospital variation of postoperative length of stay in patients with an uncomplicated course of esophageal cancer surgery. | Registry-based retrospective multicenter study. Hospital- level comparison. | Logistic regression | N=1,007 patients that underwent surgery for esophageal cancer between 2015 and 2018 at Dutch hospitals | | Length of stay | | - **Basic demographics:**  Sex, age - **Patient factors:** ASA-class, CCI, preoperative weight loss, BMI, and previous gastroesophageal surgery - **Tumor factors:**  Tumor location, T-stage, N-stage, salvage surgery, histology, histology - **Treatment factors:** Neoadjuvant therapy - **Other factors:**  Annual volume, surgical procedure, and each hospital’s complication rate during the study period | | Model described. No internal or external validation | Not reported | They showed O/E rates in a volume-funnel plot with 95 % confidence interval |
| **Author** | **Year** | **Country** | **Cancer** | **Aim** | **Study design** | **Modelling method** | **Number of cases** | | **Outcome** | | **Case-mix factors** | | **Validation** | **Performance metrics** | **Impact of  case-mix** |
| **Gray** | 2021 | UK | Breast cancer | To assess between-hospital variation in all-cause mortality and breast cancer specific mortality in patients with breast cancer | Population-based register study. Network-level comparison | Parametric regression modelling, assuming a Weibull distribution | N=48,978 patients that underwent breast cancer surgery 2001 and 2018 in all Scottish health boards | | All-cause mortality and disease-specific mortality | | - **Basic demographics:**  Age - **Patient factors:** Comorbidity burden - **Tumor factors:** Prognosis according to predict - **Socioeconomic factors:** Socioeconomic status - **Other factors:** Year of diagnosis | | No model description. No internal or external validation | Not reported | Not reported |
| **Greidanus** | 2024 | Netherlands | Colorectal cancer | To assess between hospital variation in rates of anastomotic leakage and 90-day mortality | Population-based register study. Hospital-level comparison | Logistic regression | N=44,101 patients that underwent resection for colorectal cancer between Jan 2015 and Dec 2020 at Dutch hospitals | | 60-day anastomotic leakage rate and 90-day postoperative mortality rate | | - **Basic demographics:**  Sex, age - **Patient factors:** ASA class - **Tumor factors:** Tumor stage (TNM), and urgency - **Treatment factors:**  Neoadjuvant therapy, surgical resection type, abdominal approach, defunctioning stoma created at index surgery, multivisceral resection | | Model description. No internal or external validation | Not reported | Not reported |
| **Aravani** | 2016 | UK | Colorectal cancer | To assess which factors are associated with an ideal length of hospital stay after colorectal surgery and compare rates of ideal length of stay between hospitals | Registry-based retrospective multicenter study. Hospital- level comparison | Logistic regression. | N=240,873 patients who underwent surgery for colorectal cancer at an NHS trust between 1998 and 2010 | | Length of stay. Ideal length of stay was defined as 5 days or less after the surgery | | - **Basic demographics:**  Sex, age - **Patient factors:** CCI - **Tumor factors** Tumor site, and Duke’s stage, and urgency. - **Socioeconomic factors:** Social deprivation - Treatment factors: Surgical approach - **Other factors:** Year of diagnosis | | Model described. No internal or external validation | Not reported | They showed O/E rates in a volume-funnel plot with 95 % and 99.8 % confidence intervals. On the Y-axis they multiplied the O/E-rate by the national average to get an adjusted number of ideal LOS for each hospital |
| **Author** | **Year** | **Country** | **Cancer** | **Aim** | **Study design** | **Modelling method** | **Number of cases** | | **Outcome** | | **Case-mix factors** | | **Validation** | **Performance metrics** | **Impact of  case-mix** |
| **D’Journo** | 2016 | France | Esophageal cancer | To perform external validation of the Steyerberg score for the prediction of in hospital-, 30 day and 90-day mortality after esophagectomy for cancer | Registry-based retrospective multicenter study. Hospital- level comparison | Logistic regression. | N=1,039 patients from 42 hospitals who underwent esophagectomy between 2002 and 2011 | | Inhospital-, 30-day- and 90-day mortality | | - **Basic demographics:**  Age - **Patient factors:**  Number of comorbidities - **Treatment factors:** Neoadjuvant treatment - **Other factors:** Hospital volume | | External validation | **Discrimination:** AUC  **Calibration:** Predicted to observed values in deciles of risk | They showed volume-funnel plots with absolute mortality rates on the y-axis |
| **Van den Bosch 2021** | 2021 | Netherlands | Colorectal cancer | To test whether different machine learning techniques including logistic regression predict 30-day mortality better than more simple risk scores such as Charlson Comorbidity burden and ASA | Registry-based retrospective multicenter study. Hospital- level comparison | Logistic regression, elastic net regression, random forest, and gradient boosting method | N=62,501 patients who underwent surgery for colorectal cancer between 1^st^ of January 2011 and 31^st^ of December 2016 | | 30-day mortality rates | | Case-mix variables of the logistic regression model:   - **Basic demographics:**  Sex, age - **Patient factors:** BMI, CCI, ASA score - **Tumor factors:** Preoperative tumor complications, urgency of the resection, T stage, and M stage. - **Treatment factors:** Additional resection due to metastasis or tumor ingrowth,   **For other ML-methods:** All 103 preoperative variables available in the Dutch Colorectal Cancer Register were available. | | Model described. Internal validation (data was split into a training set and a test set). No external validation | **Discrimination:** AUC | Not reported |
| **Author** | **Year** | **Country** | **Cancer** | **Aim** | **Study design** | **Modelling method** | **Number of cases** | | **Outcome** | | **Case-mix factors** | | **Validation** | **Performance metrics** | **Impact of  case-mix** |
| **Damhuis** | 2015 | Netherlands | Lung cancer | To assess hospital variation in defined outcomes | Register-based retrospective multicenter study. Hospital- level comparison | Logistic regression | N=9,579 patients who underwent lung surgery at 79 Dutch hospitals between 2005 and 2010 | | 30-day post-operative mortality and 30-day postoperative severe morbidity | | Not described which case-mix factors were tested but the final model included:   - **Basic demographics:**  Sex, age - **Treatment factors:** Type of surgery - **Other factors:** Calendar period | | Model described. No internal or external validation | Not reported | Not reported |
| **Warps** | 2021 | Netherlands | Rectal cancer | To assess hospital variation in rates of achieved textbook outcome | Population-based study. Hospital-level comparison | Logistic regression | N=20,521 patients that underwent surgery for rectal cancer between 1^st^ of January 2012 and 31^st^ of December 2019 from 76 Dutch hospitals | | Textbook outcome | | Candidate case-mix factors not reported. Included case-mix factors:   - **Basic demographics:**  Sex, age - **Patient factors:**  BMI, ASA score, CCI - **Tumor factors:** Clinical tumor stage, preoperative tumor-related complications, M-stage - **Treatment factors:** Additional resection for metastasis, multivisceral resection for local ingrowth, neoadjuvant (chemo)radiotherapy, and surgical procedure. | | Model described. No internal or external validation | Not reported | Funnel plot with resection volume on the x-axis and observed to expected outcome ratio on the Y-axis |
| **Author** | **Year** | **Country** | **Cancer** | **Aim** | **Study design** | **Modelling method** | **Number of cases** | | **Outcome** | | **Case-mix factors** | | **Validation** | **Performance metrics** | **Impact of  case-mix** |
| **Bernard** | 2023 | France | Lung cancer | To assess hospital variation in 30-day mortality rates after lung resection for lung cancer | Population-based study. Hospital- and regional level comparison | Mixed effects modelling using the fixed effects to estimate the expected hospital rates. They assigned a random intercept to each hospital | N=87,232 patients that underwent lung resection between 2013 and 2020 from 199 French hospitals | | 30-day post-operative mortality | | The candidate variables are not described in detail. The included case-mix variables were:   - **Basic demographics:**  Sex, age - **Patient factors:** Several specific comorbidities, modified CCI - **Treatment factors:** Type of pulmonary resection (the approach and extent of resection) | | No model description. No internal or external validation | Not reported | Funnel plot with O/E ratios on the Y-axis and expected rates on the X-axis |
| **Blake** | 2024 | UK | Colorectal cancer | To develop and validate a model that can be used for case-mix adjustment and risk prediction of 90-day mortality after emergency colon cancer resection | Population-based study. Hospital-level comparison | Logistic regression | N=10,578 patients who underwent emergency resection for colon cancer between 1^st^ of December 2016 and 30^th^ of November 2019 | | 90-day post-operative mortality rates | | They tested a “basic model” including the following case-mix factors:   - **Basic demographics:**  Sex, age - **Patient factors:**  ASA-grade, number of comorbidities - **Tumor factors:** T-stage, N-stage, M-stage, cancer site, emergency admission. - **Other factors:** calendar year of surgery   In the full model they added Echocardiogram, cardiac symptoms, systolic blood pressure, pulse rate, breathlessness history, Glasgow Coma Scale, urea, white blood cell count, serum creatinine, albumin, Hb, expected (by the surgeon) operative severity, expected blood loss, expected peritoneal soiling, sodium and potassium.  The chosen model included the base model plus history of breathlessness, pulse rate, systolic blood pressure, urea, albumin and sodium | | Model described. Internal validation using data splitting. No external validation | **Discrimination:** C-statistic. **Overall accuracy:**  Brier score. **Model variance explanation:** Pooled R^2^  **Calibration:** Predicted to observed values in deciles of risk | Not reported |
| **Author** | **Year** | **Country** | **Cancer** | **Aim** | **Study design** | **Modelling method** | **Number of cases** | | **Outcome** | | - **Case-mix factors** | | **Validation** | **Performance metrics** | **Impact of  case-mix** |
| **Myrdal** | 2009 | Sweden | Lung cancer | To assess geographical differences in treatment for lung cancer and secondly to assess whether these treatment disparities were associated with between-county survival differences | Register-study. County-level comparison | Cox proportional hazards modelling | N=4,375 patients diagnosed with non-small cell lung cancer between 1^st^ of January 1995 and 31^st^ of December 2003 from seven counties in one Swedish Healthcare region | | Survival | | - **Basic demographics:**  Sex, age - **Patient factors:** Smoking status - **Tumor factors:**  Histopathology, and stage - **Other factors:** Calendar period | | Model described. No internal or external validation | Not reported | Not reported |
| **Skyrud** | 2015 | Norway | Multiple cancers | To assess the impact of case-mix factors, treatment factors and place of residence on survival from all cancers combined and the six most common cancer types in Norway separately | Register-study. Regional-level comparison | Flexible parametric modelling with each home residence as a dummy variable. | N=238,821 patients that had been diagnosed with any type of cancer between 1^st^ of January 2002 and 31^st^ of December 2011 in 21 health services | | Survival | | - **Basic demographics:**  Sex, age - **Patient factors:** CCI - **Socioeconomic factors:** Educational level, and household income | | Model described. No internal or external validation. | Not reported | Not reported |
